# Interplay between psychological distress, income inequality, mental health-related medication use and consultations with a psychologist: Australian population-level data between 2011 and 2018

**DOI:** 10.1177/00048674251362049

**Published:** 2025-09-11

**Authors:** Yuan Tian, Meredith G Harris, Caley Tapp, Frances Shawyer, Graham Meadows, Joanne Enticott

**Affiliations:** 1 Monash Centre for Health Research and Implementation, Faculty of Medicine, Nursing and Health Sciences, Monash University, Melbourne, VIC, Australia; 2 Faculty of Health, Medicine and Behavioural Sciences, School of Public Health, The University of Queensland, Herston, QLD, Australia; 3 Queensland Centre for Mental Health Research, Wacol, QLD, Australia; 4 Faculty of Medicine, Nursing and Health Sciences, Department of Psychiatry, Southern Synergy, Monash University, Melbourne, VIC, Australia; 5 Centre for Mental Health, School of Population and Global Health, University of Melbourne, Melbourne, VIC, Australia; 6 School of Primary and Allied Health Care, Faculty of Medicine, Nursing and Health Sciences, Monash University, Melbourne, VIC, Australia

**Keywords:** Psychological distress, income inequality, mental health, medication, psychological care

## Abstract

**Aims:**

The aim of the study was to examine the interplay between income inequality, psychological distress, medication use and access to psychologist consultations in Australia.

**Methods:**

Hypothesis-driven secondary data analysis was conducted using nationally representative data from the 2011–2012, 2014–2015 and 2017–2018 Australian National Health Surveys. Approximately 12,000 working-age participants (18–64 years) were analysed per survey year, with subgroup interaction effects (*p* < 0.1) explored.

**Results:**

Overall, 16% of participants reported taking medications, and 5% consulted a psychologist in the past year. About 14% experienced high distress, and 5% had very-high distress in the past month. Lower-income individuals were more likely to experience high psychological distress and use mental health medications. Specifically, 30% of adults in the lowest income quintile used medications, and 14% reported very-high distress, compared to 10% and 2% in the highest income group. More low-income individuals (9%) consulted a psychologist compared to high-income individuals (4%). Interaction analyses revealed that lower-income individuals who used medication or saw a psychologist exhibited up to four times higher distress than those in higher-income groups.

**Conclusions:**

The findings reveal a concerning disparity when combined with other national data: individuals in the lowest income quintile face higher mental health symptoms, greater medication use, and are more likely to consult a psychologist, yet receive fewer consultations. This exploratory work deepens understanding of the complex relationship between income inequality, mental health symptoms, medications and healthcare utilisation in well-resourced countries like Australia. With mental ill-health rising globally, understanding these dynamics is crucial for designing equitable mental health policies.

## Introduction

### Mental health prevalence not decreasing globally

Increased mental health care spending and provision has not led, at least in Anglophone nations, to reductions in symptomatic prevalence of mental health conditions (Butterworth et al., 2020; Enticott et al., 2022; Jorm, 2018; Jorm et al., 2017; Mulder et al., 2017; Ormel et al., 2022). This Treatment Prevalence Paradox (TPP; Ormel et al., 2022) indicates complex relationships between mental health awareness, service utilisation, and outcomes, underscoring the need to better understand contributing factors to mental health burden (Foulkes and Andrews, 2023). Ideally, such research should examine population-level data to inform large-scale interventions that might ultimately improve population-level mental health (Brownson et al., 2009; Milat et al., 2014).

Possible explanations for the TPP have included persistent disparities in healthcare access based on socioeconomic status (Enticott et al., 2016), stigmatisation of mental illness hindering help-seeking (Clement et al., 2015; Reavley and Jorm, 2011), and limitations in availability or affordability of adequate treatment (Callander et al., 2017; Ngui et al., 2010), particularly for marginalised populations and those most needing it (Meadows et al., 2015; Tabatabaei-Jafari et al., 2021). These social, sociodemographic and socioeconomic determinants have been studied for decades (Allen et al., 2014; Enticott et al., 2016; Lam et al., 2019; Taylor et al., 2012). However, these do not appear to fully explain the TPP as gaps in understanding remain even after statistical adjustment for these factors. Recent discussions propose new explanations for the TPP (Furukawa and Kessler, 2019; Ormel et al., 2022; Skinner et al., 2022), highlighting potential determinants such as increased awareness (Kutcher et al., 2016) and iatrogenic influences. Recently, two potential iatrogenic influences related to depression have been proposed to help explain the paradox: ‘Oppositional Perturbation’, which relates to tolerance associated with antidepressant medications, and ‘Loss of Agency’, reflecting decreased use of self-help strategies associated with over-medicalisation of mental health issues, a potential risk when antidepressants are exclusively relied upon (Meadows et al., 2019; Ormel et al., 2022). These iatrogenic influences are plausible for other common mental disorders too and therefore require further examination for understanding the TPP more generally.

### Australian setting and opportunity

Within the Australian context, prevalence for common mental disorders at one-in-five is unchanged over the 14 years between 2007 and 2020–2021 (Australian Institute of Health and Welfare (AIHW), 2024c); while elevated psychological distress, measured as high level or above on the Kessler-10, has significantly increased from 12% to 15% between 2007 and 2018 (Enticott et al., 2022). A continuous increase in the use of antidepressants was observed from 2015 to 2021 (de Oliveira Costa et al., 2023). Combined government mental health expenditures (inflation-adjusted) have been increasing since the early 1990s, for instance at 3% per annum in real terms between 2017 and 2022, now reaching over AU$12 billion per year (AIHW, 2024a). In the context of this large and ongoing investment into mental health care, where it is estimated that more than one-in-ten Australians access mental health care annually (AIHW, 2024b), the TPP is clearly evident.

In recent decades, numerous Australian studies have examined links between socioeconomic inequality and mental health conditions (Butterworth et al., 2020; Enticott et al., 2022; Enticott et al., 2016; Isaacs et al., 2018; Lam et al., 2019; Taylor et al., 2012), mental health-related medication use (Brett et al., 2017; Butterworth et al., 2013), and psychological service utilisation (Dawadi et al., 2024; Pirkis et al., 2022). However, few have explored potential explanations for the TPP by comprehensively assessing the interplay between socioeconomic inequality, psychological distress, medication use and consultations with a psychologist over time.

To address this gap, the current study uses a large, high-quality dataset from three nationally representative Australian National Health Surveys (NHS), conducted consecutively between 2011 and 2018 by Australian Bureau of Statistics (ABS) (2013, 2017, 2019), to inform health and social policy in Australia. These surveys, spanning 7 years and based on self-reported data from the Australian population, consistently measured psychological distress and are sufficiently comparable to be combined into a single dataset (Enticott et al., 2022), increasing precision of estimates. A key innovation of this study is the use of interaction analysis to uncover how socioeconomic factors moderate the relationships between psychological distress, medication use and psychologist consultations, enabling a more nuanced understanding of the structural drivers of mental health inequalities and TPP.

The 2020–2021 NHS was excluded as a pragmatic methodological decision for several reasons. First, the 2011–2018 period reflects a relatively stable phase in Australian mental health care and policy, offering more reliable national data for exploring the complex dynamics underpinning TPP. Second, the 2020–2021 NHS coincided with the onset of the COVID-19 pandemic, which introduced major disruptions to health, economic, and mental health service delivery (Newby et al., 2020; Santomauro et al., 2021), complicating hypothesis testing in such a volatile context. Third, understanding pre-pandemic trends is essential for informing future policy, as these trends offer critical context for interpreting post-pandemic developments. Despite the pandemic’s transformative impact, mental health inequalities and socioeconomic disparities have remained persistent (AIHW, 2024d; Li et al., 2025), underscoring the importance of addressing these enduring structural issues. Finally, the 2020–2021 NHS used online rather than face-to-face data collection and did not distinguish psychologist consultations from those with psychiatrists or accredited counsellors, which limit comparability with earlier surveys (ABS, 2022).

### Aims and hypotheses

**Figure 1.**
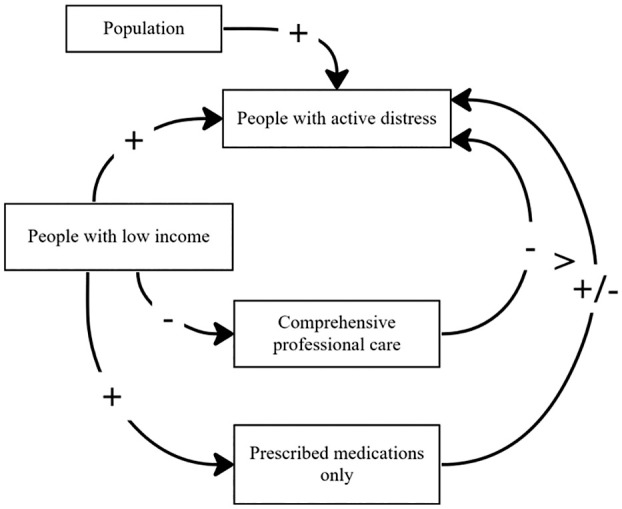
**Causal loop diagram of this study’s hypotheses**. In this study: ‘comprehensive professional care’ represents consultations with a psychologist in the past 12 months, and ‘prescribed medications only’ represents taking psycholeptics or psychoanaleptics in the past 2 weeks. Adapted from Meadows et al. (2019). ± represents positive or negative correlation along the pathway. > suggests that the left-hand pathway has greater effect than the right.

This study aims to explore the interplay between psychological distress, income inequality, mental health-related medication use and consultations with a psychologist, using 2011–2018 Australian NHS data. The causal loop diagram representing the study hypotheses in Figure 1, was informed and adapted from Meadows et al. (2019). Drawing on this we frame the following hypotheses:

People in households with lower incomes with mental health problems are

1. less likely to receive services delivered by a psychologist,
2. more likely to be prescribed medications,
3. more likely to have unresolved symptoms as indicated by elevated psychological distress.

## Methods

### Study design

Secondary analysis of nationwide, cross-sectional, representative NHS (ABS, 2013, 2017, 2019) examining three consecutive surveys over a 7-year period (2011–2018).

### Study data

In each NHS, face-to-face interviews were conducted by trained ABS interviewers with one adult resident sampled from private dwellings in urban and rural areas, using stratified multistage sampling. Information about sociodemographic status, lifestyle indicators, chronic physical conditions, mental health conditions, medication usage and physical measures were collected. In the 2011–2012, 2014–2015 and 2017–2018 NHS, 15,565, 14,723 and 16,376 households responded (rates of 84.8%, 82.0% and 76.0%, respectively). This study included 12,332, 11,296 and 12,183 participants aged 18 to 64 years from the 2011–2012, 2014–2015 and 2017–2018 NHS to represent Australia’s working-age population.

### Psychological distress

Psychological distress was measured using the Kessler Psychological Distress ten-item Scale (K10) (Kessler et al., 2002; Slade et al., 2011). The K10 contains 10 questions asking about frequencies of negative emotions in the past 4 weeks, rated on a 5-point scale: (1) all of the time, (2) most of the time, (3) some of the time, (4) a little of the time, and (5) none of the time (Kessler et al., 2002). Total score ranges from 10 (no distress) to 50 (extreme distress). Cut-off points of 22 and 30 were used in this study to identify high (22–29) and very-high (30–50) psychological distress (Andrews and Slade, 2001). The content of the K10 aligns with symptoms of affective and anxiety disorders and very-high K10 rates strongly predict recent mental disorders (American Psychological Association, 2020; Andrews and Slade, 2001). Unlike more comprehensive mental health specific surveys, the K10 is consistently collected across NHS surveys and regularly has high response rates, making a good indicator of national mental disorder trends (Atlantis et al., 2012; Butterworth et al., 2020; Enticott et al., 2022).

### Medication use

Medications taken in the last 2 weeks were grouped according to the Anatomical Therapeutic Chemical (ATC) classification (WHO Collaborating Centre for Drug Statistics Methodology (WHOCC), 2023). Psycholeptics (N05) include antipsychotics, anxiolytics, hypnotics and sedatives, while psychoanaleptics (N06) include antidepressants, psychostimulants and antidementia drugs (WHOCC, 2023).

### Household income and other sociodemographic factors

Household income levels were expressed as equivalised household income and grouped into quintiles consistent with previous research (Enticott et al., 2016; Isaacs et al., 2018). Other sociodemographic factors consisted of sex, age, country of birth, geographical location, highest educational attainment, employment status and average working hours.

### Consultations with a psychologist

Information regarding consultations with a psychologist was available in the 2011–2012 and 2014–2015 NHS (but not the 2017–2018 NHS) (Supplementary Table S7) by asking participants whether they had consulted a psychologist for health in the last 12 months (see additional data items used in supplementary data analysis in Supplementary Table S8).

### Data analysis

Analyse﻿s were conducted using Stata 17 (StataCorp, 2021). The data for all surveys were directly age-standardised based on the Australian population distributions from the 2016 Australian Census of Population and Housing data. High and high/very-high psychological distress, medication use, income inequality, other socioeconomic factors, health service use and comorbidity were provided as categorical variables. The prevalence of psychological distress and medication use was expressed as proportions.

A descriptive analysis of the prevalence of high and high/very-high psychological distress and psycholeptics and psychoanaleptics medication use in each socioeconomic, mental health service use, and physical comorbidity category was conducted. Logistic regressions examined the odds of experiencing very-high or high/very-high psychological distress and of taking medications, among people in different household income quintiles. Multivariate regressions adjusted for (a) survey year and other socioeconomic factors, including sex, age, and geographical location (Model 1), (b) medication use (for psychological distress) or psychological distress levels (for medication use) plus variables in the previous model (Model 2), (c) comorbidity plus variables in the previous model (Model 3), (d) health service use plus variables in the previous model (Model 4). Since health service data were unavailable for 2017–2018 NHS, Model 3 was the primary model.

Interaction effects were tested between household income and medication use and between household income and consultations with a psychologist. Interaction effects producing *p*-values less than 0.1 were investigated (Sun et al., 2010), and marginal effects were plotted.

### Ethical considerations

Common practice for the ABS is data collection following the Census and Statistics Act 1905. Under the ABS and Universities Australia Agreement (ABS, 2025), researchers affiliated with participating universities can access the basic, anonymized, microdata for 2011–2012, 2014–2015, and 2017–2018 NHS. Therefore, ethics approval was not required for our analyses.

## Results

Overall, 4.6% (95% CI = [4.4, 4.8]) of participants reported very-high psychological distress, 13.5% [13.1, 13.9] experienced high/very-high psychological distress, 4.8% [4.5, 5.0] took psycholeptics, 13.0% [12.6, 13.4] used psychoanaleptics, 15.5% [15.0, 15.9] took psycholeptics and/or psychoanaleptics, and 4.9% [4.6, 5.2] consulted a psychologist (Table 1).

**Table 1.** Age-standardised prevalence of psychological distress, mental health-related medication use (psycholeptics and psychoanaleptics), and consultations with a psychologist in the Australian working-age population, 2011-2012, 2014-2015, and 2017-2018^$^.

			Very-high psychological distress	High/very-high psychological distress	Psycholeptics Medication Use	Psychoanaleptics Medication Use	Consulted a Psychologist for Health in the Last 12 Months^§^
		Total %	Rate% (95% CI)	Rate% (95% CI)	Rate% (95% CI)	Rate% (95% CI)	Rate% (95% CI)
Household Income Quintile	1 (Low)	13.9	13.9 (12.9, 15.0)	29.8 (28.4, 31.2)	11.8 (10.7, 13.0)	23.5 (22.0, 25.1)	8.6 (7.5, 9.7)
	2	14.4	8.2 (7.4, 9.0)	20.9 (19.7, 22.1)	9.4 (8.4, 10.4)	20.3 (18.9, 21.8)	5.8 (5.0, 6.8)
	3	20.8	3.4 (3.0, 3.9)	12.0 (11.2, 12.8)	3.4 (2.8, 3.9)	13.1 (12.1, 14.2)	3.9 (3.3, 4.5)
	4	24.9	1.8 (1.5, 2.1)	8.6 (8.0, 9.3)	2.6 (2.2, 3.1)	10.6 (9.8, 11.5)	4.3 (3.8, 4.9)
	5 (High)	25.9	1.6 (1.3, 1.9)	6.2 (5.7, 6.8)	2.5 (2.1, 3.0)	8.8 (8.1, 9.6)	4.3 (3.8, 4.9)
Year^%^	2011 - 12	34.5	3.9 (3.6, 4.3)	11.8 (11.2, 12.4)	4.6 (4.1, 5.0)	11.2 (10.5, 11.9)	3.3 (3.0, 3.7)
	2014 - 15	31.6	4.6 (4.2, 5.0)	13.2 (12.5, 13.8)	4.7 (4.2, 5.1)	13.4 (12.7, 14.2)	6.6 (6.2, 7.1)
	2017 - 18	33.9	5.4 (5.0, 5.8)	15.6 (14.9, 16.3)	5.1 (4.6, 5.6)	14.6 (13.8, 15.4)	NA
Age Group	18 - 24	14.9	5.0 (4.3, 5.8)	16.5 (15.3, 17.8)	3.3 (2.6, 4.2)	10.4 (9.0, 11.8)	4.7 (3.9, 5.6)
	25 - 34	23.1	3.6 (3.1, 4.0)	12.2 (11.5, 13.0)	3.3 (2.8, 3.9)	10.6 (9.8, 11.5)	4.9 (4.4, 5.6)
	35 - 44	21.5	3.9 (3.5, 4.3)	12.1 (11.4, 12.8)	5.4 (4.8, 6.0)	13.1 (12.2, 14.0)	5.8 (5.2, 6.4)
	45 - 54	21.3	5.7 (5.2, 6.2)	14.1 (13.3, 14.8)	5.6 (5.1, 6.3)	15.5 (14.6, 16.5)	5.2 (4.7, 5.9)
	55 - 64	19.2	5.1 (4.7, 5.7)	13.6 (12.8, 14.4)	5.9 (5.4, 6.5)	15.1 (14.2, 16.0)	3.7 (3.2, 4.3)
Sex^%^	Male	47.0	3.7 (3.4, 4.0)	11.1 (10.6, 11.6)	4.8 (4.4, 5.2)	10.1 (9.5, 10.7)	3.5 (3.1, 3.8)
	Female	53.0	5.5 (5.1, 5.8)	15.7 (15.2, 16.2)	4.8 (4.4, 5.2)	15.2 (14.6, 15.8)	6.2 (5.8, 6.6)
Location^%^	Major cities	65.0	4.4 (4.1, 4.7)	13.1 (12.7, 13.6)	4.4 (4.1, 4.8)	12.1 (11.6, 12.7)	5.1 (4.8, 5.5)
	Inner regional	17.9	5.5 (4.9, 6.1)	15.1 (14.1, 16.0)	6.5 (5.7, 7.2)	16.4 (15.2, 17.5)	5.4 (4.7, 6.1)
	Other	17.1	4.5 (4.0, 5.1)	13.4 (12.5, 14.3)	4.2 (3.6, 4.8)	13.0 (11.9, 14.1)	3.6 (3.0, 4.2)
Psycholeptics Medication Use in the Last 2 Weeks^%^	Yes	4.8	30.1 (27.1, 33.2)	55.6 (52.3, 58.9)	NA	NA	29.2 (25.4, 33.0)
	No	95.2	4.5 (4.3, 4.8)	14.0 (13.5, 14.5)	NA	NA	5.3 (5.0, 5.7)
Psychoanaleptics Medication Use in the Last 2 Weeks^%^	Yes	13.0	21.0 (19.5, 22.6)	45.6 (43.8, 47.5)	NA	NA	25.5 (23.4, 27.6)
	No	87.0	3.5 (3.2, 3.7)	11.6 (11.1, 12.1)	NA	NA	3.8 (3.4, 4.1)
Consulted a Psychologist for Health in the Last 12 Months^%, §^	Yes	4.9	22.5 (20.0, 25.0)	47.1 (44.1, 50.0)	19.9 (17.3, 22.4)	48.1 (44.9, 51.2)	NA
	No	95.1	3.3 (3.1, 3.6)	10.7 (10.3, 11.1)	3.6 (3.3, 3.9)	9.8 (9.4, 10.3)	NA
Consulted a GP for Health in the Last 12 Months^%, §^	Yes	84.4	4.8 (4.5, 5.1)	13.6 (13.1, 14.1)	5.0 (4.6, 5.3)	13.4 (12.8, 13.9)	5.8 (5.5, 6.1)
	No	15.5	1.4 (1.0, 1.7)	5.9 (5.2, 6.7)	1.0 (0.6, 1.5)	1.9 (1.2, 2.5)	0.3 (0.1, 0.5)

CI: confidence interval; NA, not available $. All cell sizes had > 50 observations. %. The prevalence of psychological distress and mental health-related medication use were directly age-standardised to 2016 Australian Census, which consists of a total of *n* = 146,585 survey participants aged 18–64 years. §. Data related to health service use presented in this table was only available from 2011–2012 and 2014–2015 NHS. The 2017–2018 NHS did not include questions about health service use.

Regarding household income disparities, 29.6% [28.0, 31.3] in the lowest quintile and 10.3% [9.5, 11.1] in the highest quintile took psycholeptics and/or psychoanaleptics medications, while 8.6% [7.5, 9.7] and 4.3% [3.8, 4.9] consulted a psychologist, respectively. In the lowest and highest income quintiles, 21.9% [20.1, 23.8] and 7.9% [6.6, 8.3] took medications but did not consult a psychologist, 3.4% [2.6, 4.3] and 2.8% [2.3, 3.4] consulted a psychologist but did not take medications, and 7.1% [6.0, 8.3] and 2.3% [1.9, 2.9] accessed both interventions, respectively (Table 2). See other results in Supplementary Table S1 & S6.

**Table 2.** Age-standardised prevalence of psychological distress among cross-tabulation between household income, medication use, and health service use in the Australian working-age population, 2011-2012, 2014-2015, and 2017-2018^$^.

			Very-high psychological distress	High/very-high psychological distress
		Total %	Rate% (95% CI)^%^	Rate%^%^ (95% CI)^%^
Psycholeptics and/or Psychoanaleptics Medication Use in the Last 2 Weeks	Yes	15.5	20.9 (19.5, 22.3)	45.1 (43.4, 46.8)
	No	84.5	3.0 (2.7, 3.2)	10.7 (10.3, 11.1)
Consulted a Psychologist for Health in the Last 12 Months^§^	Yes	4.9	22.5 (20.0, 25.0)	47.1 (44.1, 50.0)
	No	95.1	3.3 (3.1, 3.6)	10.7 (10.3, 11.1)
Only Took Psycholeptics and/or Psychoanaleptics Medications and Did Not Consult a Psychologist^§^	Yes	11.2	16.8 (14.9, 18.7)	39.2 (36.7, 41.8)
	No	88.8	3.8 (3.5, 4.2)	11.6 (11.0, 12.1)
Only Consulted a Psychologist^§^ and Did Not Take Psycholeptics and/or Psychoanaleptics Medications	Yes	2.9	13.2 (10.0, 16.5)	36.8 (32.3, 41.4)
	No	97.1	5.1 (4.7, 5.4)	14.0 (13.4, 14.6)
Both Took Psycholeptics and/or Psychoanaleptics Medications and Consulted a Psychologist^§^	Yes	3.5	31.8 (27.8, 35.9)	59.1 (54.8, 63.3)
	No	96.5	4.3 (4.0, 4.7)	13.1 (12.5, 13.7)
By Household Income Quintile				
Used Psycholeptics and/or Psychoanaleptics Medication in the Last 2 Weeks	1 (Low)	29.6	34.5 (31.4, 37.8)	60.5 (57.2, 63.7)
	2	24.9	23.4 (20.4, 26.6)	51.0 (47.4, 54.7)
	3	14.8	15.8 (13.0, 19.0)	36.8 (33.0, 40.8)
	4	12.0	9.4 (7.2, 12.0)	30.8 (27.1, 34.6)
	5 (High)	10.3	9.3 (7.0, 12.0)	26.7 (23.1, 30.6)
Consult a Psychologist in the Last 12 Months^§^	1 (Low)	8.6	42.3 (35.8, 49.1)	68.0 (61.5, 74.1)
	2	5.8	24.1 (17.6, 31.5)	55.7 (47.6, 63.6)
	3	3.8	19.4 (13.5, 26.5)	45.8 (37.8, 54.0)
	4	4.3	11.5 (7.5, 16.7)	37.0 (30.4, 44.0)
	5 (High)	4.3	8.8 (5.4, 13.4)	22.7 (17.3, 28.9)
Only Took Psycholeptics and/or Psychoanaleptics Medications and Did Not Consult a Psychologist^§^	1 (Low)	21.9	30.1 (25.7, 34.7)	54.4 (49.5, 59.3)
	2	19.8	19.7 (15.8, 24.0)	45.6 (40.5, 50.7)
	3	11.5	13.7 (10.1, 18.0)	33.6 (28.5, 39.1)
	4	8.5	7.6 (4.7, 11.3)	26.6 (21.5, 32.2)
	5 (High)	7.4	7.0 (4.3, 10.8)	24.8 (19.8, 30.4)
Only Consulted a Psychologist^§^ and Did Not Take Psycholeptics and/or Psychoanaleptics Medications	1 (Low)	3.4	36.9 (25.3, 49.8)	58.5 (45.6, 70.6)
	2	2.8	16.1 (7.6, 28.3)	51.8 (38.0, 65.3)
	3	2.5	13.0 (6.1, 23.3)	36.2 (25.0, 48.7)
	4	2.6	5.9 (1.9, 13.2)	28.2 (19.0, 39.0)
	5 (High)	2.8	2.9 (0.6, 8.4)	14.7 (8.5, 23.1)
Both Took Psycholeptics and/or Psychoanaleptics Medications and Consulted a Psychologist^§^	1 (Low)	7.1	46.7 (38.0, 55.4)	74.8 (66.6, 81.9)
	2	4.6	32.2 (22.6, 43.1)	65.5 (54.6, 75.4)
	3	2.5	28.2 (18.1, 40.1)	54.9 (42.7, 66.8)
	4	2.8	17.2 (10.2, 26.4)	48.4 (37.9, 59.0)
	5 (High)	2.3	15.7 (8.6, 25.3)	32.5 (22.7, 43.7)
Did Not Take Psycholeptics or Psychoanaleptics Medication in the Last 2 Weeks	1 (Low)	70.4	9.4 (8.2, 10.7)	23.5 (21.7, 25.4)
	2	75.1	5.7 (4.8, 6.7)	16.6 (15.1, 18.2)
	3	85.2	2.4 (2.0, 3.0)	10.2 (9.2, 11.2)
	4	88.0	1.1 (0.8, 1.5)	7.1 (6.4, 7.9)
	5 (High)	89.7	1.0 (0.7, 1.3)	4.8 (4.2, 5.4)
Did Not Consult a Psychologist in the Last 12 Months^§^	1 (Low)	91.4	10.8 (9.6, 12.1)	24.3 (22.6, 26.1)
	2	94.2	6.4 (5.5, 7.4)	17.4 (15.9, 18.9)
	3	96.2	2.5 (2.1, 3.1)	9.8 (8.8, 10.7)
	4	95.7	1.2 (0.9, 1.6)	6.8 (6.0, 7.5)
	5 (High)	95.7	1.0 (0.8, 1.4)	4.7 (4.1, 5.4)
Did Not Take Psycholeptics and/or Psychoanaleptics Medications nor Consulted a Psychologist^§^	1 (Low)	67.6	8.0 (6.6, 9.6)	19.7 (17.5, 21.9)
	2	72.9	4.6 (3.6, 5.8)	14.1 (12.4, 16.0)
	3	83.4	1.8 (1.3, 2.4)	8.2 (7.1, 9.4)
	4	86.1	0.7 (0.5, 1.1)	5.6 (4.8, 6.5)
	5 (High)	87.5	0.8 (0.5, 1.2)	3.7 (3.1, 4.4)

CI, confidence interval. $. All cell sizes had > 50 observations. %. The prevalence of psychological distress was directly age-standardised to the 2016 Australian Census, which consists of a total of *n* = 146,585 survey participants aged 18–64 years. §. Data related to health service use presented in this table was only available from 2011–2012 and 2014–2015 NHS. The 2017–2018 NHS did not include questions about health service use.

### Psychological distress

Participants in the lowest household income quintile had significantly higher rates of very-high (13.9% [12.9, 15.0]) and high/very-high (29.8% [28.4, 31.2]) psychological distress in the past 4 weeks, respectively, compared to those in the highest income quintile (1.6% [1.3, 1.9] and 6.2% [5.7, 6.8]) (Table 1). Adjusting for year, socioeconomic factors, medication use, and physical comorbidity (Model 3; Table 3), multivariate regression suggested that participants in the first three income quintiles were significantly more likely to experience very-high psychological distress than those in the fifth/highest quintile (*p* < 0.001), with the lowest quintile showing the highest odds ratio (OR) of 6.1 [4.8, 7.7]. Similarly, a statistically significant correlation was observed for high/very-high psychological distress across the first four income quintiles (*p* < 0.001), with the lowest quintile again showing the highest OR of 4.8 [4.2, 5.6]. In additional regression models (Supplementary Tables S2.1-S2.3 & S4), this relationship remained statistically significant for both very-high and high/very-high psychological distress (*p* < 0.001).

**Table 3.** Odds ratio of psychological distress unadjusted and adjusted (**Model 3**: for year, sex, age, geographical location, household income, psycholeptics use, psychoanaleptics use, and physical comorbidity) for the Australian working-age population, 2011–2012, 2014–2015, and 2017–2018.

		Univariate Analysis				Multivariate Analysis (Model 3)			
		Very-high Psychological Distress		High/Very-high Psychological Distress		Very-high Psychological Distress		High/Very-high Psychological Distress	
		Unadjusted OR (95% CI)	*p*-value	Unadjusted OR (95% CI)	*p*-value	Adjusted OR (95% CI)	*p*-value	Adjusted OR (95% CI)	*p*-value
Household Income Quintile	1 (Low)	10.1 (8.3, 12.3)	< 0.001***	6.4 (5.7, 7.2)	< 0.001***	6.1 (4.8, 7.7)	< 0.001***	4.8 (4.2, 5.6)	< 0.001***
	2	5.5 (4.5, 6.8)	< 0.001***	4.0 (3.5, 4.5)	< 0.001***	3.7 (2.9, 4.8)	< 0.001***	3.3 (2.8, 3.8)	< 0.001***
	3	2.2 (1.7, 2.7)	< 0.001***	2.1 (1.8, 2.3)	< 0.001***	2.2 (1.7, 2.8)	< 0.001***	2.0 (1.8, 2.4)	< 0.001***
	4	1.1 (0.9, 1.5)	0.294	1.4 (1.3, 1.6)	< 0.001***	1.0 (0.8, 1.4)	0.755	1.4 (1.2, 1.6)	< 0.001***
	5 (High)	(Ref)		(Ref)		(Ref)		(Ref)	
Year	2011/12	(Ref)		(Ref)		(Ref)		(Ref)	
	2014/15	1.1 (1.0, 1.3)	0.044*	1.1 (1.0, 1.2)	0.003**	0.9 (0.8, 1.1)	0.282	1.0 (0.9, 1.1)	0.671
	2017/18	1.4 (1.2, 1.5)	< 0.001***	1.4 (1.3, 1.5)	< 0.001***	1.0 (0.9, 1.2)	0.736	1.2 (1.1, 1.3)	< 0.001***
Sex	Male	(Ref)		(Ref)		(Ref)		(Ref)	
	Female	1.5 (1.3, 1.6)	< 0.001***	1.5 (1.4, 1.6)	< 0.001***	1.1 (0.9, 1.2)	0.301	1.1 (1.0, 1.2)	0.200
Age Group	18 - 24	(Ref)		(Ref)		(Ref)		(Ref)	
	25 - 34	0.7 (0.6, 0.8)	< 0.001***	0.7 (0.6, 0.8)	< 0.001***	0.6 (0.4, 0.7)	< 0.001***	0.6 (0.5, 0.7)	< 0.001***
	35 - 44	0.8 (0.6, 0.9)	0.004**	0.7 (0.6, 0.8)	< 0.001***	0.5 (0.4, 0.7)	< 0.001***	0.5 (0.4, 0.6)	< 0.001***
	45 - 54	1.1 (1.0, 1.4)	0.142	0.8 (0.7, 0.9)	0.001**	0.5 (0.4, 0.6)	< 0.001***	0.4 (0.3, 0.5)	< 0.001***
	55 - 64	1.0 (0.9, 1.2)	0.790	0.8 (0.7, 0.9)	< 0.001***	0.3 (0.2, 0.4)	< 0.001***	0.3 (0.2, 0.3)	< 0.001***
Location	Major Cities	(Ref)		(Ref)		(Ref)		(Ref)	
	Inner Regional	1.3 (1.1, 1.4)	< 0.001***	1.2 (1.1, 1.3)	< 0.001***	0.9 (0.7, 1.0)	0.097	0.9 (0.8, 1.0)	0.005**
	Other	1.0 (0.9, 1.2)	0.541	1.0 (1.0, 1.1)	0.389	0.9 (0.8, 1.1)	0.408	0.9 (0.8, 1.1)	0.368
Psycholeptics Medication Use	No	(Ref)		(Ref)		(Ref)		(Ref)	
	Yes	9.0 (7.8, 10.3)	< 0.001***	7.8 (6.9, 8.8)	< 0.001***	2.9 (2.5, 3.5)	< 0.001***	3.1 (2.6, 3.6)	< 0.001***
Psychoanaleptics Medication Use	No	(Ref)		(Ref)		(Ref)		(Ref)	
	Yes	7.3 (6.5, 8.1)	< 0.001***	6.3 (5.8, 6.8)	< 0.001***	2.9 (2.6, 3.4)	< 0.001***	3.1 (2.8, 3.5)	< 0.001***
Comorbid Physical Conditions (Current and Long-Term)	No Comorbidity	(Ref)		(Ref)		(Ref)		(Ref)	
	Only 1	2.0 (1.5, 2.7)	< 0.001***	1.6 (1.4, 1.8)	< 0.001***	2.6 (1.7, 4.0)	< 0.001***	1.8 (1.5, 2.2)	< 0.001***
	2 - 4	4.7 (3.8, 6.0)	< 0.001***	3.0 (2.7, 3.3)	< 0.001***	4.9 (3.4, 7.1)	< 0.001***	3.0 (2.5, 3.5)	< 0.001***
	5 +	26.2 (21.0, 32.7)	< 0.001***	11.6 (10.5, 12.9)	< 0.001***	16.2 (11.2, 23.4)	< 0.001***	8.7 (7.4, 10.2)	< 0.001***

*** <0.001, **<0.01, and *<0.05. CI, confidence interval; OR, odds ratio. Model 3 was adjusted for year, sex, age, geographical location, household income, psycholeptics use, psychoanaleptics use and physical comorbidity.

### Medication use

Participants in the lowest income quintile had the highest prevalence of psycholeptics (11.8% [10.7, 13.0]) and psychoanaleptics (23.5% [22.0, 25.1]) intake in the last 2 weeks; those in the highest quintile reported the lowest rates of 2.5% [2.1, 3.0] and 8.8% [8.1, 9.6], respectively (Table 1). Differences were statistically significant, with ORs of 2.0 ([1.6, 2.5], *p* < 0.001) for psycholeptics use and 1.3 ([1.2, 1.6], *p* < 0.001) for psychoanaleptics use, after adjusting for year, socioeconomic factors, psychological distress, and physical comorbidity (Model 3; Table 4). Regression models indicated that lower income individuals were significantly more likely to use these medications, with significance persisting across the first two income quintiles for psycholeptics (*p* < 0.001) and the first three for psychoanaleptics (*p* < 0.05) (Table 4 and Supplementary Tables S3.1-S3.3 & S4).

**Table 4.** Odds ratio of mental health-related medication use (psycholeptics and psychoanaleptics) unadjusted and adjusted (**Model 3**: for year, sex, age, geographical location, household income, K10 category, and physical comorbidity) for the Australian working-age population, 2011–2012, 2014–2015, and 2017–2018.

		Univariate Analysis				Multivariate Analysis (Model 3)			
		Psycholeptics Medication Use		Psychoanaleptics Medication Use		Psycholeptics Medication Use		Psychoanaleptics Medication Use	
		Unadjusted OR (95% CI)	*p*-value	Unadjusted OR (95% CI)	*p*-value	Adjusted OR (95% CI)	*p*-value	Adjusted OR (95% CI)	*p*-value
Household Income Quintile	1 (Low)	5.2 (4.3, 6.4)	< 0.001***	3.2 (2.8, 3.6)	< 0.001***	2.0 (1.6, 2.5)	< 0.001***	1.3 (1.2, 1.6)	< 0.001***
	2	4.0 (3.3, 5.0)	< 0.001***	2.6 (2.3, 3.0)	< 0.001***	2.0 (1.6, 2.5)	< 0.001***	1.4 (1.2, 1.7)	< 0.001***
	3	1.4 (1.1, 1.7)	0.013**	1.6 (1.4, 1.8)	< 0.001***	1.0 (0.8, 1.3)	0.963	1.2 (1.0, 1.4)	0.017*
	4	1.0 (0.8, 1.3)	0.706	1.2 (1.1, 1.4)	0.002**	0.9 (0.7, 1.2)	0.551	1.1 (1.0, 1.3)	0.150
	5 (High)	(Ref)		(Ref)		(Ref)		(Ref)	
Year	2011/12	(Ref)		(Ref)		(Ref)		(Ref)	
	2014/15	1.0 (0.9, 1.2)	0.788	1.2 (1.1, 1.4)	< 0.001***	0.9 (0.7, 1.0)	0.105	1.2 (1.0, 1.3)	0.009**
	2017/18	1.1 (1.0, 1.3)	0.179	1.3 (1.2, 1.5)	< 0.001***	0.9 (0.7, 1.0)	0.094	1.1 (1.0, 1.2)	0.140
Sex	Male	(Ref)		(Ref)		(Ref)		(Ref)	
	Female	1.0 (0.9, 1.1)	0.878	1.6 (1.4, 1.7)	< 0.001***	0.9 (0.7, 1.0)	0.025*	1.4 (1.3, 1.5)	< 0.001***
Age Group	18 - 24	(Ref)		(Ref)		(Ref)		(Ref)	
	25 - 34	1.0 (0.7, 1.3)	0.985	1.0 (0.9, 1.2)	0.744	1.4 (1.0, 2.0)	0.071	1.3 (1.1, 1.6)	0.015*
	35 - 44	1.7 (1.3, 2.2)	< 0.001***	1.3 (1.1, 1.5)	0.002**	1.9 (1.4, 2.7)	< 0.001***	1.4 (1.1, 1.7)	0.002**
	45 - 54	1.7 (1.3, 2.3)	< 0.001***	1.6 (1.3, 1.9)	< 0.001***	1.5 (1.1, 2.2)	0.019*	1.3 (1.1, 1.6)	0.015*
	55 - 64	1.8 (1.4, 2.4)	< 0.001***	1.5 (1.3, 1.8)	< 0.001***	1.5 (1.1, 2.2)	0.018*	1.2 (0.9, 1.4)	0.165
Location	Major Cities	(Ref)		(Ref)		(Ref)		(Ref)	
	Inner Regional	1.5 (1.3, 1.7)	< 0.001***	1.4 (1.3, 1.6)	< 0.001***	1.2 (1.0, 1.4)	0.027*	1.2 (1.1, 1.4)	< 0.001***
	Other	1.0 (0.8, 1.1)	0.738	1.1 (1.0, 1.2)	0.091	0.8 (0.7, 1.0)	0.034*	1.0 (0.9, 1.1)	0.778
K10 Category	Low	(Ref)		(Ref)		(Ref)		(Ref)	
	Moderate	3.4 (2.8, 4)	< 0.001***	3.2 (2.9, 3.5)	< 0.001***	2.3 (1.9, 2.7)	< 0.001***	2.5 (2.3, 2.8)	< 0.001***
	High	8.6 (7.2, 10.2)	< 0.001***	7.4 (6.6, 8.2)	< 0.001***	4.5 (3.7, 5.5)	< 0.001***	4.5 (4.0, 5.1)	< 0.001***
	Very-high	20.4 (17.2, 24.3)	< 0.001***	14.5 (12.8, 16.4)	< 0.001***	8.2 (6.6, 10.1)	< 0.001***	6.9 (6.0, 8.0)	< 0.001***
Comorbid Physical Conditions (Current and Long-Term)	No Comorbidity	(Ref)		(Ref)		(Ref)		(Ref)	
	Only 1	1.8 (1.3, 2.5)	0.001**	2.7 (2.2, 3.3)	< 0.001***	1.5 (1.0, 2.2)	0.037*	2.4 (1.9, 3.0)	< 0.001***
	2 - 4	4.0 (3.1, 5.3)	< 0.001***	5.7 (4.8, 6.8)	< 0.001***	2.8 (2.1, 3.9)	< 0.001***	4.5 (3.7, 5.5)	< 0.001***
	5 +	14.4 (11.0, 18.8)	< 0.001***	16.2 (13.6, 19.2)	< 0.001***	5.6 (4.0, 7.7)	< 0.001***	8.1 (6.6, 9.9)	< 0.001***

*** <0.001, **<0.01, and *<0.05. CI, confidence interval; K10, the Kessler Psychological Distress Scale; OR, odds ratio. Model 3 was adjusted for year, sex, age, geographical location, household income, K10 category and physical comorbidity.

### Consultations with a psychologist

Among people consulting a psychologist, 22.5% [20.0, 25.0] and 47.1% [44.1, 50.0] experienced very-high and high/very-high psychological distress, respectively, compared to those who did not consult (3.3% [3.1, 3.6] and 10.7% [10.3, 11.1]) (Table 1), with adjusted ORs of 2.3 [1.8, 2.8] and 2.6 [2.2, 3.1] (*p* < 0.001; Supplementary Table S2.3). In addition, 19.9% [17.3, 22.4] and 48.1% [44.9, 51.2] of participants consulting a psychologist took psycholeptics and psychoanaleptics, respectively (Table 1), compared to those who did not consult (3.6% [3.3, 3.9] and 9.8% [9.4, 10.3]), with adjusted ORs of 2.2 [1.8, 2.8] and 3.6 [3.1, 4.3] (*p* < 0.001; Supplementary Table S3.3).

Between the lowest and highest income quintiles, results (Table 2) revealed 3.7- and 4.8-fold discrepancies in the prevalence of very-high psychological distress for participants taking psycholeptics and/or psychoanaleptics medications (34.5% [31.4, 37.8] versus 9.3% [7.0, 12.0]) and those consulting a psychologist (42.3% [35.8, 49.1] versus 8.8% [5.4, 13.4]). The income-related gap widened for individuals who consulted a psychologist without taking medications (36.9% [25.3, 49.8] versus 2.9% [0.6, 8.4]) or who only took medications without consulting (30.1% [25.7, 34.7] versus 7.0% [4.3, 10.8]). The gap persisted among people who accessed both medications and psychologist consultations (46.7% [38.0, 55.4] versus 15.7% [8.6, 25.3]), who did not take medications (9.4% [8.2, 10.7] versus 1.0% [0.7, 1.3]), or who did not consult a psychologist (10.8% [9.6, 12.1] versus 1.0% [0.8, 1.4]).

### Interaction effects between household income, medication use and consultations with a psychologist

Effects on very-high distress rates were statistically significant for the interaction between household income and psycholeptics and/or psychoanaleptics medication use (*p* = 0.0011), with the interaction being significant for participants in the first- and second-income quintiles who took medications (coefficient = −0.7 and −0.7, *p* = 0.002 and 0.002) compared to the fifth/highest quintile for those not taking medications (Supplementary Table S5.1–S5.3). For the interaction between household income and consultations with a psychologist, significant interaction effects on very-high distress rates were present (*p* = 0.04) and were significant for the second income quintile among those who consulted a psychologist (coefficient = −0.7, *p* = 0.044) (Supplementary Table S5.4–S5.5).

**Figure 2.**
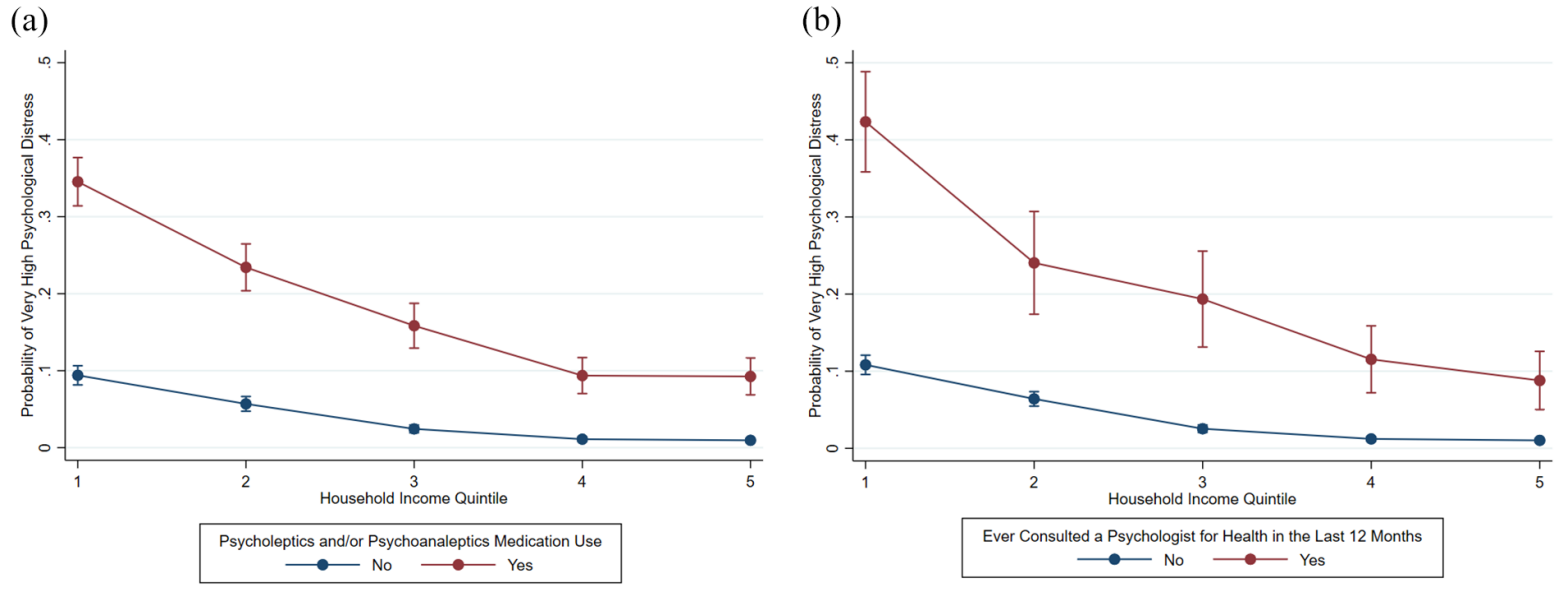
Adjusted predictions on the probability of very-high psychological distress based on (a) household income and mental health-related medication use (psycholeptics and/or psychoanaleptics) in the last 2 weeks and (b) household income and consultations with a psychologist for health in the last 12 months, for the Australian working-age population, 2011–2012, 2014–2015, and 2017–2018^$, %^. $. Figure 2(b) only included working-aged participants from the 2011–2012 and 2014–2015 NHS, because data related to health service use presented in Figure 2 (b) was only available from the 2011–2012 and 2014–2015 NHS. The 2017–2018 NHS did not include questions about health service use. %. The interaction effects were both statistically significant for household income and mental health-related medication use (psycholeptics and/or psychoanaleptics) in the last 2 weeks (*p* = 0.0011) (Figure 2(a)) and household income and consultations with a psychologist for health in the last 12 months (*p* = 0.0391) (Figure 2(b)) on the prevalence of very-high psychological distress.

Marginal effects demonstrated a fourfold difference in the probability of experiencing very-high distress in the lowest income quintile, for those taking medications (0.35 [0.31, 0.38] versus 0.09 [0.07, 0.12]) or consulting a psychologist (0.42 [0.36, 0.49] versus 0.09 [0.05, 0.13]), compared to the highest quintile (Supplementary Table S5.3–S5.6). In Figure 2(a), the gap in very-high distress probability between those taking and not taking medications narrowed as household income increased, across the first four income quintiles. Similarly, in Figure 2(b), the gap in very-high distress risks narrowed with higher income between those who consulted a psychologist and those who did not.

Similar significant interaction effects were observed for very-high distress (*p* = 0.0635). The gap in marginal effects between the lowest and highest income quintiles was more evident among individuals relying solely on medications (0.30 [0.26, 0.34] versus 0.07 [0.04, 0.10]), compared to those accessing both medications and psychologist consultations (0.47 [0.38, 0.55] versus 0.16 [0.08, 0.23]) (Supplementary Table/Figure S5.7–S5.10).

### Time trends

Overall increases of 1.5% and 3.8% in very-high and high/very-high psychological distress rates were observed between 2011–2012 and 2017–2018, while the changes were 0.5% and 3.4% for psycholeptics and psychoanaleptics use, respectively (Supplementary Table/ Material S9).

## Discussion

Between 2011 and 2018, approximately 14% of Australian adults experienced high/very-high psychological distress, with 5% reporting very-high distress in the past month, and about 16% taking mental health medications. From 2011 to 2015, approximately 5% consulted a psychologist in the past year.

Thirty percent of people in the lowest income quintile and 10% of the highest took medications, supporting hypothesis 2. Distress was higher among lower income individuals with 30% reporting high distress and 14% very-high distress compared to 6% and 2% in the highest income quintile. Interaction effects confirmed significantly greater distress among lower income medication users and/or psychologist consultees. Thus, hypothesis 3 gains support.

Going against hypothesis 1, findings showed 9% in the lowest income quintile and 4% in the highest consulted a psychologist in the past year. These national surveys recorded if a person consulted a psychologist in the past year but not how often, as did convergent census linkage findings (ABS, 2011). There are much higher numbers of overall consultations in wealthier areas (Meadows et al., 2015; Pirkis et al., 2022), for example, in 2019, there were 260 psychology services per 1000 people in the least disadvantaged areas by quintile and 140 in the most disadvantaged (Dawadi et al., 2024). Thus, this current study, in conjunction with previous research, shows while more low-income individuals had seen a psychologist annually, overall consultation numbers mean that high-income individuals on average received longer or more intensive courses of treatment, potentially more comprehensive and effective. This new evidence is important for Australian mental health services planning, and further research is needed to examine the frequency, duration and quality of mental health care received by individuals across different socioeconomic groups.

Lower income groups may be less likely to complete full treatment courses due to complex factors, with out-of-pocket costs being a key barrier. Nationally, 25.6% of Australians needing psychological care delayed or avoided the service due to cost (28.7% for psychiatrists) (ABS, 2023–24). Consequently, lower income individuals may be more likely, on financial grounds, to disengage from psychological treatment prematurely, potentially leading to demoralisation – with possible iatrogenic psychological effects (Meadows et al., 2019). In this case, wealthier individuals may typically benefit from comprehensive psychological treatment, while financially disadvantaged individuals may receive harm.

Our study examined psychologist consultation data from mid-2011 to mid-2014. A more recent analysis linking Medicare Benefits Schedule records with other administrative and survey data from 2018 to 2021 (Pirkis et al., 2022) found that Australians in the lowest income quintile were less likely to access the Better Access services primarily delivered by psychologists, following a mental health treatment plan, compared to those in the highest income quintile. The rate differences remained to be around 10% from 2018 to 2021. Their findings, primarily based on administrative data, provide a clear illustration of a persistent disparity in access to specialised psychological care across socioeconomic groups, which complements our results derived from population-level self-reported survey data and aligns with earlier evidence syntheses. Future research could benefit from examining mental health service use patterns over time through the integration of multiple data sources.

The other findings in this study were consistent with other research using national survey data in terms of the significant influence of income inequality on psychological distress in Australia (Enticott et al., 2022; Isaacs et al., 2018). As similar to this study, Enticott et al. (2022) observed a large gap between the lowest and highest household income quintiles in very-high psychological distress rates (1.0% [95% CI: 0.6, 1.5] versus 10.9% [9.3, 12.5]) with an OR of 11.5 ([9.9, 13.4], *p* < 0.001), based on NHS data from 2001 to 2017–2018. Furthermore, Isaacs et al. (2018) discovered a similar statistically significant relationship between lower household income and very-high psychological distress rates via analysis on the 2011–2012 NHS, with around eight times greater chances to experience very-high distress for people in the lowest income quintile compared to the highest. The strong correlation between income inequality and medication use in our study also supported past literature on the same topic (Butterworth et al., 2013; Tanana et al., 2022). In Butterworth et al.’s study (2013), antidepressant use was statistically significantly correlated to socioeconomic measures such as unemployment and financial hardship with an overall OR of 2.9 ([2.1, 4.1], *p* < 0.05). In addition, Tanana et al. (2022) found significantly higher odds of psychotropic medications prescription for the most socioeconomically disadvantaged paediatric patients (OR = 1.2 [1.1, 1.3], *p* < 0.001), compared to the most advantaged patients.

Furthermore, interaction analysis revealed that low-income Australians who relied solely on medications in the past year were more likely to experience very-high psychological distress than their high-income counterparts, and this income-related gap was larger compared to Australians who accessed both medications and psychologist consultations. Although cross-sectional data used in this study could not provide evidence of causality, results might imply that the over-reliance on pharmaceutical therapies might have produced higher rates of distress among participants on lower incomes than those on higher incomes. This might be because individuals with lower incomes may have limited access to non-pharmaceutical interventions (Bernard et al., 2023; Corscadden et al., 2018), such as psychotherapy or lifestyle support, and are more likely to experience financial strain and other stressors that exacerbate distress (Kiely et al., 2015), leading to a heavier reliance on medications for managing their mental health symptoms. Moreover, psychological treatment and medication alone are insufficient to alleviate, and may even exacerbate, financial hardship and cost-of-living stress (Callander and Schofield, 2018; Rohatgi et al., 2021). If this interpretation holds, it would provide evidence for the above mechanisms proposed to underlie the TPP. Given the significantly larger percentages of acute psychological distress in the past 4 weeks among those who consulted a psychologist in the past year compared to those who did not, it is possible that the current professional mental health care may have limitations in relieving acute symptoms. Alternatively, this pattern may reflect a tendency for distressed individuals to seek psychological support. Future research, preferably using longitudinal data, is warranted to explore potential reverse causality between psychological treatment utilisation and mental health symptoms among low-income populations.

In addition, our findings might lend plausibility to the hypotheses in relation to the linkage between psychological distress, mental health related medication use, and consultations with a psychologist. Our findings, taken together with the available Medicare data (Dawadi et al., 2024; Pirkis et al., 2022), suggest that people on lower incomes were likely to be relying on psycholeptics and psychoanaleptics medications to mitigate their mental health symptoms, and such reliance of medicalised therapies among people on low incomes failed to alleviate their distress which resulted in relapse and greater percentages of low-income individuals experiencing distress (Andrews et al., 2011). Further discussion of the TPP is available in Supplementary Material S10-11.

### Limitations

A limitation of the study is the use of data from 2011–2018; however, as discussed, this period offered a stable pre-pandemic context for examining the study’s hypotheses, and the exclusion of the 2020–2021 NHS reflects a deliberate methodological choice due to the disruptive impact of COVID-19. Considering the impact of the COVID-19 pandemic on the TPP in Australia, we anticipate that new causal loop diagrams and DAGs will be required, informed by literature, expert inputs, and consensus on the complex inferential pathways – and this paper presents a solid foundation for that future work. Another limitation was restricted comparability between the surveys, meaning not all information was directly comparable, including ‘consulting a psychologist’ was not available in the 2017–2018 NHS (Supplementary Table S7). Also, information about seeing a psychologist was not directly asked in the NHS but was provided in a list of possible health professions that the participants may have consulted for health reasons, and a psychiatrist was not included in the given list, which might influence responses. Moreover, since the NHS was conducted every 3 years transversely rather than longitudinally, we were not able to obtain a longitudinal cohort therefore making the findings exploratory.

## Conclusions

This causal loop-informed, epidemiological health research uniquely investigates the interplay between psychological distress, income inequality, medication use and psychologist consultations using nationally representative data. These findings show that more low-income individuals took medications, yet their distress rates were significantly higher, supporting the TPP in Australia whereby the treatments received by individuals in different income groups were not associated with comparable distress-level findings. The main findings, that the lower income quintile is taking more medications and more report consulting a psychologist, combined with other high-quality data reporting that they are receiving less frequent psychologist follow-up consultations relative to need, together support our casual loop diagram and study hypotheses such that mental health symptoms in the form of elevated distress are significantly greater in those with the lowest incomes. This work deepens our understanding of the complex relationship between income inequality, mental health symptoms, medications and healthcare utilisation in well-resourced countries like Australia. Given the growing burden of mental ill-health globally, understanding the interplay between these factors is critical to design effective public health and other mental health interventions. Population-level strategies to support more frequent comprehensive mental health care (Malhi et al., 2021), less reliance on medications, and reduced inequality are essential to mitigate disparities evident in Australian mental health and psychological distress landscape. We hope this analysis will serve as an exemplar to inspire future researchers to continue this crucial work, enabling evidence-informed policies to more effectively address pressing and seemingly unsolvable issues such as those regarding population mental health and service provisions.

## Supplemental Material

sj-docx-1-anp-10.1177_00048674251362049 – Supplemental material for Interplay between psychological distress, income inequality, mental health-related medication use and consultations with a psychologist: Australian population-level data between 2011 and 2018 Supplemental material, sj-docx-1-anp-10.1177_00048674251362049 for Interplay between psychological distress, income inequality, mental health-related medication use and consultations with a psychologist: Australian population-level data between 2011 and 2018 by Yuan Tian, Meredith G Harris, Caley Tapp, Frances Shawyer, Graham Meadows and Joanne Enticott in Australian & New Zealand Journal of Psychiatry
