# Supplementary material for: Interplay between psychological distress, income inequality, mental health-related medication use and consultations with a psychologist: Australian population-level data between 2011 and 2018
Source: Aust N Z J Psychiatry. 2025 Sep 11;59(11):989–1003. doi: 10.1177/00048674251362049 (PMC12569130; doi:10.1177/00048674251362049)
Supplement: sj-docx-1-anp-10.1177_00048674251362049 – Supplemental material for Interplay between psychological distress, income inequality, mental health-related medication use and consultations with a psychologist: Australian population-level data between 2011 and 2018 [file sj-docx-1-anp-10.1177_00048674251362049.docx]

**Supplementary Materials**

Table of Contents

[**Supplementary Table S1.** Age-standardised prevalence of psychological distress, mental health-related medication use (psycholeptics and psychoanaleptics), and consultations with a psychologist, for sociodemographic factors, K10 category, service use, mental disorders, and comorbidity in the Australian working age population, 2011/12, 2014/15, and 2017/18^†^ 3](#_Toc177033641)

[**Supplementary Table S2.1.** Odds ratio of psychological distress unadjusted and adjusted (**Model 1**: for year, sex, age, geographical location, and household income) for the Australian working age population, 2011-12, 2014-15, and 2017-18 5](#_Toc177033642)

[**Supplementary Table S2.2.** Odds ratio of psychological distress unadjusted and adjusted (**Model 2**: for year, sex, age, geographical location, household income, psycholeptics use, and psychoanaleptics use) for the Australian working age population, 2011-12, 2014-15, and 2017-18 6](#_Toc177033643)

[**Supplementary Table S2.3.** Odds ratio of psychological distress unadjusted and adjusted (**Model 4**: for year, sex, age, geographical location, household income, psycholeptics use, psychoanaleptics use, physical comorbidity, whether consulted a GP, and whether consulted a psychologist) for the Australian working age population, 2011-12, and 2014-15**^a^** 7](#_Toc177033644)

[**Supplementary Table S3.1.** Odds ratio of mental health-related medication use (psycholeptics and psychoanaleptics) unadjusted and adjusted (**Model 1**: for year, sex, age, geographical location, and household income) for the Australian working age population, 2011-12, 2014-15, and 2017-18 9](#_Toc177033645)

[**Supplementary Table S3.2.** Odds ratio of mental health-related medication use (psycholeptics and psychoanaleptics) unadjusted and adjusted (**Model 2**: for year, sex, age, geographical location, household income, and K10 category) for the Australian working age population, 2011-12, 2014-15, and 2017-18 10](#_Toc177033646)

[**Supplementary Table S3.3.** Odds ratio of mental health-related medication use (psycholeptics and psychoanaleptics) unadjusted and adjusted (**Model 4**: for year, sex, age, geographical location, household income, K10 category, physical comorbidity, whether consulted a GP, and whether consulted a psychologist) for the Australian working age population, 2011-12, and 2014-15 11](#_Toc177033647)

[**Supplementary Table S4.** Univariate odds ratio of psychological distress and medication use (psycholeptics and psychoanaleptics) for the Australian working age population, 2011/12, 2014/15, and 2017/18 13](#_Toc177033648)

[**Interaction effects:** 15](#_Toc177033649)

[**Supplementary Table S5.1.** Likelihood-ratio test for logistic regression model of psychological distress, with household income and mental health-related medication use (psycholeptics and/or psychoanaleptics) in the last 2 weeks as predictors, with interaction effects, for the Australian working age population, 2011-12, 2014-15, and 2017-18 15](#_Toc177033650)

[**Supplementary Table S5.2.** Logistic regression model of psychological distress with household income and mental health-related medication use (psycholeptics and/or psychoanaleptics) in the last 2 weeks as predictors and with interaction effects, for the Australian working age population, 2011-12, 2014-15, and 2017-18 15](#_Toc177033651)

[**Supplementary Table S5.3.** Marginal effects of household income and mental health-related medication use (psycholeptics and/or psychoanaleptics) on very-high psychological distress with interaction effects, for the Australian working age population, 2011-12, 2014-15, and 2017-18 16](#_Toc177033652)

[**Supplementary Table S5.4.** Likelihood-ratio test for logistic regression model of psychological distress, with household income and consultations with a psychologist in the last 12 months on psychological distress as predictors, with interaction effects, for the Australian working age population, 2011-12, and 2014-15 16](#_Toc177033653)

[**Supplementary Table S5.5.** Logistic regression model of psychological distress with household income and consultations with a psychologist in the last 12 months as predictors and with interaction effects, for the Australian working age population, 2011-12, and 2014-15 17](#_Toc177033654)

[**Supplementary Table S5.6.** Marginal effects of household income and consultations with a psychologist in the last 12 months on very-high psychological distress with interaction effects, for the Australian working age population, 2011-12, and 2014-15 17](#_Toc177033655)

[**Supplementary Table S5.7.** Likelihood-ratio test for logistic regression model of psychological distress, with household income and mental health-related medication use (psycholeptics and/or psychoanaleptics) in the last 2 weeks plus consultations with a psychologist in the last 12 months as predictors, with interaction effects, for the Australian working age population, 2011-12, and 2014-15 18](#_Toc177033656)

[**Supplementary Table S5.8.** Logistic regression model of psychological distress with household income and mental health-related medication use (psycholeptics and/or psychoanaleptics) in the last 2 weeks plus consultations with a psychologist in the last 12 months as predictors and with interaction effects, for the Australian working age population, 2011-12, and 2014-15 18](#_Toc177033657)

[**Supplementary Table S5.9.** Marginal effects of household income and mental health-related medication use (psycholeptics and/or psychoanaleptics) in the last 2 weeks plus consultations with a psychologist in the last 12 months on very-high psychological distress with interaction effects, for the Australian working age population, 2011-12, and 2014-15 19](#_Toc177033658)

[**Supplementary Figure S5.10.** Adjusted predictions of household income and mental health-related medication use (psycholeptics and/or psychoanaleptics) in the last 2 weeks plus consultations with a psychologist in the last 12 months on the probability for very-high psychological distress, among the Australian working age population, 2011-12, and 2014-15^†, ‡^ 20](#_Toc177033659)

[**Supplementary Table S6.** Number of participants in each variable category^†^ 21](#_Toc177033660)

[**Supplementary Table S7.** Comparability of variables between the 2011-12, 2014-15, and 2017-18 National Health Surveys (NHS) 24](#_Toc177033661)

[**Supplementary Table S8.** Additional data items used in supplementary data analysis 27](#_Toc177033662)

[**Supplementary Material S9.** Details about the results related to time trend and sociodemographic factors 28](#_Toc177033663)

[Time trend 28](#_Toc177033664)

[Sociodemographic factors 28](#_Toc177033665)

[**Supplementary Material S10.** Treatment Prevalence Paradox (TPP) 29](#_Toc177033666)

[**Supplementary Material S11.** Possible responses 30](#_Toc177033667)

[**References:** 31](#_Toc177033668)

**Supplementary Table S1.** Age-standardised prevalence of psychological distress, mental health-related medication use (psycholeptics and psychoanaleptics), and consultations with a psychologist, for sociodemographic factors, K10 category, service use, mental disorders, and comorbidity in the Australian working age population, 2011/12, 2014/15, and 2017/18^†^

|  |  |  | **Very-high psychological distress** | **High/very-high psychological distress** | **Psycholeptics Medication Use** | **Psychoanaleptics Medication Use** | **Consulted a Psychologist for Health** |
| --- | --- | --- | --- | --- | --- | --- | --- |
|  |  | **Total %** | **Rate^a^% (95% CI)** | **Rate^a^% (95% CI)** | **Rate^a^% (95% CI)** | **Rate^a^% (95% CI)** | **Rate^a^% (95% CI)** |
| **Australian Born^a^** | Yes | 72.2 | 4.9 (4.6, 5.2) | 14.2 (13.8, 14.7) | 5.3 (5.0, 5.6) | 14.8 (14.3, 15.3) | 5.6 (5.3, 6.0) |
|  | No | 27.8 | 3.9 (3.5, 4.3) | 11.5 (10.8, 12.1) | 3.4 (2.9, 3.8) | 7.9 (7.2, 8.5) | 3.0 (2.6, 3.4) |
| **Completed Tertiary Education^a^** | Yes | 66.2 | 3.6 (3.4, 3.9) | 11.6 (11.1, 12.0) | 3.8 (3.5, 4.1) | 11.9 (11.3, 12.4) | 5.2 (4.9, 5.6) |
|  | No | 33.8 | 6.8 (6.3, 7.3) | 17.8 (17.1, 18.5) | 7.1 (6.5, 7.7) | 15.9 (15.0, 16.8) | 4.3 (3.8, 4.7) |
| **Employment Status^a^** | Employed | 77.1 | 2.4 (2.3, 2.6) | 9.6 (9.2, 9.9) | 2.6 (2.4, 2.8) | 10.1 (9.6, 10.5) | 4.0 (3.7, 4.2) |
|  | Unemployed | 3.7 | 13.4 (11.4, 15.4) | 30.9 (28.2, 33.6) | 9.0 (7.0, 11) | 21.0 (18.2, 23.9) | 8.9 (6.9, 10.9) |
|  | Not in the Labour Force | 19.2 | 12.3 (11.4, 13.1) | 26.7 (25.6, 27.8) | 12.7 (11.7, 13.7) | 23.1 (21.8, 24.3) | 8.2 (7.3, 9.1) |
| **Hours Working/Week^a^** | Not in the Labour Force | 19.2 | 12.3 (11.4, 13.1) | 26.7 (25.6, 27.8) | 12.7 (11.7, 13.7) | 23.1 (21.8, 24.3) | 8.2 (7.3, 9.1) |
|  | 24 hours or less | 14.6 | 4.2 (3.6, 4.8) | 14.0 (13.1, 15.0) | 4.3 (3.7, 5.0) | 14.1 (13.0, 15.3) | 6.4 (5.5, 7.2) |
|  | 25 - 39 hours | 26.3 | 2.5 (2.1, 2.8) | 9.6 (9.0, 10.2) | 2.5 (2.1, 2.8) | 10.8 (10.0, 11.6) | 4.1 (3.6, 4.6) |
|  | 40 - 49 hours | 22.3 | 1.8 (1.5, 2.1) | 7.6 (6.9, 8.2) | 2.0 (1.6, 2.4) | 8.2 (7.4, 8.9) | 3.0 (2.5, 3.4) |
|  | 50 hours or more | 14.0 | 2.0 (1.5, 2.5) | 8.3 (7.4, 9.2) | 2.2 (1.5, 2.8) | 6.9 (6.0, 7.9) | 2.9 (2.3, 3.5) |
| **K10 Category^a^** | Low | 64.4 | NA | NA | 1.5 (1.3, 1.7) | 5.6 (5.2, 6.0) | 2.0 (1.7, 2.2) |
|  | Moderate | 20.9 | NA | NA | 5.1 (4.5, 5.7) | 16.2 (15.3, 17.2) | 6.4 (5.7, 7.1) |
|  | High | 8.8 | NA | NA | 11.9 (10.7, 13.2) | 31.0 (29.2, 32.9) | 14.6 (13.0, 16.2) |
|  | Very-high | 4.6 | NA | NA | 24.7 (22.3, 27.0) | 47.4 (44.6, 50.1) | 25.9 (23.1, 28.7) |
| **Time Since Last Consulted GP for Own Health^a, b^** | Never/Less than 3 months ago | 34.3 | 6.6 (6.1, 7.0) | 17.1 (16.4, 17.8) | 6.6 (6.1, 7.1) | 17.2 (16.4, 18.0) | 7.8 (7.3, 8.3) |
|  | 3 - 6 months | 13.3 | 1.9 (1.5, 2.3) | 8.9 (8.1, 9.7) | 2.1 (1.6, 2.6) | 7.4 (6.4, 8.3) | 3.1 (2.6, 3.6) |
|  | 6 - 9 months | 5.4 | 1.8 (1.2, 2.5) | 6.3 (5.2, 7.4) | 1.5 (0.8, 2.3) | 3.7 (2.5, 4.8) | 2.4 (1.7, 3.1) |
|  | 9 - 12 months | 2.8 | 1.1 (0.4, 1.8) | 6.6 (5.0, 8.1) | 1.3 (0.4, 2.1) | 2.0 (0.9, 3.1) | 1.4 (0.7, 2.2) |
|  | 1 - 2 years | 5.6 | 1.1 (0.6, 1.6) | 6.0 (5.0, 7.1) | 1.5 (0.7, 2.2) | 2.2 (1.3, 3.2) | 0.4 (0.1, 0.7) |
|  | More than 2 years | 4.4 | 1.7 (1.1, 2.4) | 5.7 (4.5, 6.8) | 0.4 (0.0, 0.9) | 1.2 (0.3, 2.1) | 0.2 (0.0, 0.4) |
| **Mood Disorders (Current and Long-Term)^a, c, d^** | Yes | 14.4 | 25.4 (23.9, 27.0) | 55.6 (53.9, 57.4) | 17.6 (16.2, 19.0) | 54.5 (52.6, 56.5) | 29.5 (27.1, 31.9) |
|  | No | 85.6 | 1.6 (1.4, 1.7) | 7.6 (7.2, 8.0) | 2.1 (1.9, 2.4) | 5.1 (4.7, 5.5) | 3.2 (2.8, 3.5) |
| **Anxiety Related Problems (Current and Long-Term)^a, c, e^** | Yes | 11.9 | 20.8 (19.5, 22.1) | 46.2 (44.6, 47.8) | 15.5 (14.2, 16.8) | 42.6 (40.8, 44.3) | 24.1 (22.3, 25.9) |
|  | No | 88.1 | 2.0 (1.8, 2.3) | 8.6 (8.2, 9.0) | 2.4 (2.1, 2.7) | 7.2 (6.8, 7.7) | 2.9 (2.7, 3.1) |
| **Mood and Anxiety Related Disorders (Current and Long-Term)^a, c, f^** | Yes | 21.5 | 18.8 (17.7, 19.9) | 44.7 (43.2, 46.1) | 14.6 (13.5, 15.7) | 45.4 (43.8, 46.9) | 25.6 (23.8, 27.4) |
|  | No | 78.5 | 1.2 (1.0, 1.3) | 6.2 (5.8, 6.5) | 1.4 (1.2, 1.7) | 2.7 (2.4, 3.0) | 1.9 (1.6, 2.2) |
| **Comorbid Physical Conditions (Current and Long-Term)^a^** | No comorbidity | 33.6 | 0.7 (0.6, 0.9) | 4.3 (3.9, 4.7) | 1.0 (0.7, 1.3) | 2.5 (2.1, 2.9) | 1.2 (0.9, 1.4) |
|  | Only 1 | 18.7 | 1.6 (1.3, 1.9) | 7.6 (7.0, 8.3) | 1.8 (1.4, 2.2) | 6.8 (6.0, 7.6) | 2.6 (2.2, 3.1) |
|  | 2 - 4 | 31.8 | 4.3 (3.8, 4.7) | 15.0 (14.2, 15.7) | 4.3 (3.8, 4.8) | 14.1 (13.3, 14.9) | 6.2 (5.6, 6.8) |
|  | 5+ | 15.9 | 20.1 (18.7, 21.4) | 42.4 (40.8, 44) | 13.6 (12.5, 14.8) | 31.9 (30.3, 33.5) | 18.7 (16.9, 20.4) |

CI, confidential interval; K10, the Kessler Psychological Distress Scale; NA, not available. †. All cell sizes had >50 observations.

a. The prevalence of psychological distress and mental health-related medication use were direct age-standardised to 2016 Australian Census, which consists of a total of n = 146,585 survey participants aged 18-64 years.

b. Data related to health service use presented in this table was only available from the 2011-12 and 2014-15 NHS. The 2017-18 NHS did not include questions about health service use.

c. Data related to the mental health conditions presented in this table was only from the 2014-15 and 2017-18 NHS, since these mental health conditions were directly comparable between the 2014-15 and 2017-18 NHS but not comparable between 2011-12 and 2014-15 NHS.

d. Conditions recorded in the “Mood Disorders” variable included “feeling depressed”, “other mood (affective) disorders” and “depression”, which were classified based on the 10^th^ Revision of the International Classification of Diseases (ICD10).

e. Conditions recorded in the “Anxiety Related Problems” variable included “feeling anxious, nervous or tense”, “anxiety disorders”, “panic disorders/panic attacks”, “phobic anxiety disorders”, “post-traumatic stress disorder”, and “obsessive compulsive disorder”, which were classified based on the 10^th^ Revision of the International Classification of Diseases (ICD10).

f. Conditions recorded in the “Mood and Anxiety Related Disorders” variable included mood disorders, such as “feeling depressed”, “other mood (affective) disorders” and “depression”, and anxiety related problems, such as “feeling anxious, nervous or tense”, “anxiety disorders”, “panic disorders/panic attacks”, “phobic anxiety disorders”, “post-traumatic stress disorder”, and “obsessive compulsive disorder”. These conditions were classified based on the 10^th^ Revision of the International Classification of Diseases (ICD10).

**Supplementary Table S2.1.** Odds ratio of psychological distress unadjusted and adjusted (**Model 1**: for year, sex, age, geographical location, and household income) for the Australian working age population, 2011-12, 2014-15, and 2017-18

|  |  | **Univariate Analysis** | | | | **Multivariate Analysis (Model 1)** | | | |
| --- | --- | --- | --- | --- | --- | --- | --- | --- | --- |
|  |  | **Very-high Psychological Distress** | | **High/Very-high Psychological Distress** | | **Very-high Psychological Distress** | | **High/Very-high Psychological Distress** | |
|  |  | **Unadjusted OR (95% CI)** | **p-value** | **Unadjusted OR (95% CI)** | **p-value** | **Adjusted OR (95% CI)** | **p-value** | **Adjusted OR (95% CI)** | **p-value** |
| **Year** | 2011/12 | (Ref) |  | (Ref) |  | (Ref) |  | (Ref) |  |
|  | 2014/15 | 1.1 (1.0, 1.3) | 0.044* | 1.1 (1.0, 1.2) | 0.003** | 1.1 (0.9, 1.2) | 0.416 | 1.1 (1.0, 1.2) | 0.031* |
|  | 2017/18 | 1.4 (1.2, 1.5) | <0.001*** | 1.4 (1.3, 1.5) | <0.001*** | 1.2 (1.1, 1.4) | 0.001** | 1.3 (1.2, 1.4) | 0.001** |
| **Sex** | Male | (Ref) |  | (Ref) |  | (Ref) |  | (Ref) |  |
|  | Female | 1.5 (1.3, 1.6) | <0.001*** | 1.5 (1.4, 1.6) | <0.001*** | 1.4 (1.2, 1.5) | <0.001*** | 1.4 (1.3, 1.5) | <0.001*** |
| **Age Group** | 18 - 24 | (Ref) |  | (Ref) |  | (Ref) |  | (Ref) |  |
|  | 25 – 34 | 0.7 (0.6, 0.8) | <0.001*** | 0.7 (0.6, 0.8) | <0.001*** | 0.8 (0.6, 1.0) | 0.041* | 1.1 (1.0, 1.3) | 0.069 |
|  | 35 - 44 | 0.8 (0.6, 0.9) | 0.004** | 0.7 (0.6, 0.8) | <0.001*** | 0.9 (0.7, 1.1) | 0.18 | 0.9 (0.8, 0.9) | 0.003** |
|  | 45 – 54 | 1.1 (1.0, 1.4) | 0.142 | 0.8 (0.7, 0.9) | 0.001** | 1.2 (1.0, 1.5) | 0.057 | 0.9 (0.8, 0.9) | 0.002** |
|  | 55 - 64 | 1.0 (0.9, 1.2) | 0.79 | 0.8 (0.7, 0.9) | <0.001*** | 0.9 (0.7, 1.1) | 0.387 | 0.8 (0.7, 0.9) | <0.001*** |
| **Location** | Major Cities | (Ref) |  | (Ref) |  | (Ref) |  | (Ref) |  |
|  | Inner Regional | 1.3 (1.1, 1.4) | <0.001*** | 1.2 (1.1, 1.3) | <0.001*** | 1.1 (0.9, 1.2) | 0.39 | 1.0 (0.9, 1.1) | 0.638 |
|  | Other | 1.0 (0.9, 1.2) | 0.541 | 1.0 (1.0, 1.1) | 0.389 | 0.9 (0.8, 1.1) | 0.484 | 1.0 (0.9, 1.0) | 0.291 |
| **Household Income Quintile** | 1 (Low) | 10.1 (8.3, 12.3) | <0.001*** | 6.4 (5.7, 7.2) | <0.001*** | 9.7 (7.9, 11.8) | <0.001*** | 6.2 (5.5, 7.0) | <0.001*** |
|  | 2 | 5.5 (4.5, 6.8) | <0.001*** | 4.0 (3.5, 4.5) | <0.001*** | 5.3 (4.3, 6.6) | <0.001*** | 3.8 (3.4, 4.3) | <0.001*** |
|  | 3 | 2.2 (1.7, 2.7) | <0.001*** | 2.1 (1.8, 2.3) | <0.001*** | 2.2 (1.7, 2.7) | <0.001*** | 2.0 (1.8, 2.3) | <0.001*** |
|  | 4 | 1.1 (0.9, 1.5) | 0.294 | 1.4 (1.3, 1.6) | <0.001*** | 1.1 (0.9, 1.5) | <0.001*** | 1.4 (1.2, 1.6) | <0.001*** |
|  | 5 (High) | (Ref) |  | (Ref) |  | (Ref) |  | (Ref) |  |

***<0.001, **<0.01, and *<0.05. CI, confidential interval; OR, odds ratio. Model 1 was adjusted for year, sex, age, geographical location, and household income.

**Supplementary Table S2.2.** Odds ratio of psychological distress unadjusted and adjusted (**Model 2**: for year, sex, age, geographical location, household income, psycholeptics use, and psychoanaleptics use) for the Australian working age population, 2011-12, 2014-15, and 2017-18

|  |  | **Univariate Analysis** | | | | **Multivariate Analysis (Model 2)** | | | |
| --- | --- | --- | --- | --- | --- | --- | --- | --- | --- |
|  |  | **Very-high Psychological Distress** | | **High/Very-high Psychological Distress** | | **Very-high Psychological Distress** | | **High/Very-high Psychological Distress** | |
|  |  | **Unadjusted OR (95% CI)** | **p-value** | **Unadjusted OR (95% CI)** | **p-value** | **Adjusted OR (95% CI)** | **p-value** | **Adjusted OR (95% CI)** | **p-value** |
| **Year** | 2011/12 | (Ref) |  | (Ref) |  | (Ref) |  | (Ref) |  |
|  | 2014/15 | 1.1 (1.0, 1.3) | 0.044* | 1.1 (1.0, 1.2) | 0.003** | 1.0 (0.8, 1.1) | 0.656 | 1.0 (0.9, 1.1) | 0.654 |
|  | 2017/18 | 1.4 (1.2, 1.5) | <0.001*** | 1.4 (1.3, 1.5) | <0.001*** | 1.1 (1.0, 1.3) | 0.097 | 1.3 (1.2, 1.4) | <0.001*** |
| **Sex** | Male | (Ref) |  | (Ref) |  | (Ref) |  | (Ref) |  |
|  | Female | 1.5 (1.3, 1.6) | <0.001*** | 1.5 (1.4, 1.6) | <0.001*** | 1.2 (1.0, 1.3) | 0.025* | 1.2 (1.1, 1.3) | 0.001** |
| **Age Group** | 18 - 24 | (Ref) |  | (Ref) |  | (Ref) |  | (Ref) |  |
|  | 25 – 34 | 0.7 (0.6, 0.8) | <0.001*** | 0.7 (0.6, 0.8) | <0.001*** | 0.6 (0.5, 0.8) | <0.001*** | 0.6 (0.5, 0.8) | 0.001** |
|  | 35 - 44 | 0.8 (0.6, 0.9) | 0.004** | 0.7 (0.6, 0.8) | <0.001*** | 0.7 (0.5, 0.9) | 0.003** | 0.6 (0.5, 0.7) | 0.001** |
|  | 45 – 54 | 1.1 (1.0, 1.4) | 0.142 | 0.8 (0.7, 0.9) | 0.001** | 0.8 (0.6, 1.1) | 0.143 | 0.7 (0.6, 0.8) | 0.001** |
|  | 55 - 64 | 1.0 (0.9, 1.2) | 0.79 | 0.8 (0.7, 0.9) | <0.001*** | 0.6 (0.4, 0.7) | <0.001*** | 0.5 (0.4, 0.6) | 0.001** |
| **Location** | Major Cities | (Ref) |  | (Ref) |  | (Ref) |  | (Ref) |  |
|  | Inner Regional | 1.3 (1.1, 1.4) | <0.001*** | 1.2 (1.1, 1.3) | <0.001*** | 0.9 (0.8, 1.1) | 0.511 | 0.9 (0.8, 1.0) | 0.102 |
|  | Other | 1.0 (0.9, 1.2) | 0.541 | 1.0 (1.0, 1.1) | 0.389 | 1.0 (0.8, 1.1) | 0.563 | 1.0 (0.9, 1.1) | 0.485 |
| **Household Income Quintile** | 1 (Low) | 10.1 (8.3, 12.3) | <0.001*** | 6.4 (5.7, 7.2) | <0.001*** | 7.6 (6.1, 9.6) | <0.001*** | 5.6 (4.9, 6.4) | <0.001*** |
|  | 2 | 5.5 (4.5, 6.8) | <0.001*** | 4.0 (3.5, 4.5) | <0.001*** | 4.2 (3.3, 5.4) | <0.001*** | 3.5 (3.1, 4.1) | <0.001*** |
|  | 3 | 2.2 (1.7, 2.7) | <0.001*** | 2.1 (1.8, 2.3) | <0.001*** | 2.3 (1.7, 2.9) | <0.001*** | 2.0 (1.8, 2.4) | <0.001*** |
|  | 4 | 1.1 (0.9, 1.5) | 0.294 | 1.4 (1.3, 1.6) | <0.001*** | 1.1 (0.8, 1.4) | 0.567 | 1.4 (1.2, 1.6) | <0.001*** |
|  | 5 (High) | (Ref) |  | (Ref) |  | (Ref) |  | (Ref) |  |
| **Psycholeptics Medication Use** | No | (Ref) |  | (Ref) |  | (Ref) |  | (Ref) |  |
|  | Yes | 9.0 (7.8, 10.3) | <0.001*** | 7.8 (6.9, 8.8) | <0.001*** | 4.0 (3.4, 4.7) | <0.001*** | 4.1 (3.5, 4.7) | <0.001*** |
| **Psychoanaleptics Medication Use** | No | (Ref) |  | (Ref) |  | (Ref) |  | (Ref) |  |
|  | Yes | 7.3 (6.5, 8.1) | <0.001*** | 6.3 (5.8, 6.8) | <0.001*** | 4.4 (3.9, 5.0) | <0.001*** | 4.5 (4.1, 4.9) | <0.001*** |

***<0.001, **<0.01, and *<0.05. CI, confidential interval; OR, odds ratio. Model 2 was adjusted for year, sex, age, geographical location, household income, psycholeptics use, and psychoanaleptics use.

**Supplementary Table S2.3.** Odds ratio of psychological distress unadjusted and adjusted (**Model 4**: for year, sex, age, geographical location, household income, psycholeptics use, psychoanaleptics use, physical comorbidity, whether consulted a GP, and whether consulted a psychologist) for the Australian working age population, 2011-12, and 2014-15**^a^**

|  |  | **Univariate Analysis** | | | | **Multivariate Analysis (Model 4)^a^** | | | |
| --- | --- | --- | --- | --- | --- | --- | --- | --- | --- |
|  |  | **Very-high Psychological Distress** | | **High/Very-high Psychological Distress** | | **Very-high Psychological Distress** | | **High/Very-high Psychological Distress** | |
|  |  | **Unadjusted OR (95% CI)** | **p-value** | **Unadjusted OR (95% CI)** | **p-value** | **Adjusted OR (95% CI)** | **p-value** | **Adjusted OR (95% CI)** | **p-value** |
| **Year** | 2011/12 | (Ref) |  | (Ref) |  | (Ref) |  | (Ref) |  |
|  | 2014/15 | 1.1 (1.0, 1.3) | 0.044* | 1.1 (1.0, 1.2) | 0.003** | 0.8 (0.7, 1.0) | 0.046* | 0.9 (0.8, 1.0) | 0.1 |
|  | 2017/18 | 1.4 (1.2, 1.5) | <0.001*** | 1.4 (1.3, 1.5) | <0.001*** | NA |  | NA |  |
| **Sex** | Male | (Ref) |  | (Ref) |  | (Ref) |  | (Ref) |  |
|  | Female | 1.5 (1.3, 1.6) | <0.001*** | 1.5 (1.4, 1.6) | <0.001*** | 1.2 (1.0, 1.4) | 0.095 | 1.1 (1.0, 1.2) | 0.138 |
| **Age Group** | 18 - 24 | (Ref) |  | (Ref) |  | (Ref) |  | (Ref) |  |
|  | 25 – 34 | 0.7 (0.6, 0.8) | <0.001*** | 0.7 (0.6, 0.8) | <0.001*** | 0.6 (0.4, 0.9) | 0.008** | 0.7 (0.5, 0.8) | <0.001*** |
|  | 35 - 44 | 0.8 (0.6, 0.9) | 0.004** | 0.7 (0.6, 0.8) | <0.001*** | 0.7 (0.5, 1.0) | 0.031* | 0.6 (0.5, 0.7) | <0.001*** |
|  | 45 – 54 | 1.1 (1.0, 1.4) | 0.142 | 0.8 (0.7, 0.9) | 0.001** | 0.6 (0.4, 0.8) | 0.002** | 0.4 (0.3, 0.6) | <0.001*** |
|  | 55 - 64 | 1.0 (0.9, 1.2) | 0.79 | 0.8 (0.7, 0.9) | <0.001*** | 0.3 (0.2, 0.5) | <0.001*** | 0.3 (0.2, 0.3) | <0.001*** |
| **Location** | Major Cities | (Ref) |  | (Ref) |  | (Ref) |  | (Ref) |  |
|  | Inner Regional | 1.3 (1.1, 1.4) | <0.001*** | 1.2 (1.1, 1.3) | <0.001*** | 0.9 (0.7, 1.1) | 0.404 | 0.9 (0.8, 1.0) | 0.07 |
|  | Other | 1.0 (0.9, 1.2) | 0.541 | 1.0 (1.0, 1.1) | 0.389 | 0.9 (0.7, 1.1) | 0.235 | 0.9 (0.8, 1.0) | 0.133 |
| **Household Income Quintile** | 1 (Low) | 10.1 (8.3, 12.3) | <0.001*** | 6.4 (5.7, 7.2) | <0.001*** | 7.0 (5.1, 9.4) | <0.001*** | 5.3 (4.4, 6.4) | <0.001*** |
|  | 2 | 5.5 (4.5, 6.8) | <0.001*** | 4.0 (3.5, 4.5) | <0.001*** | 3.8 (2.8, 5.2) | <0.001*** | 3.6 (3.0, 4.3) | <0.001*** |
|  | 3 | 2.2 (1.7, 2.7) | <0.001*** | 2.1 (1.8, 2.3) | <0.001*** | 2.3 (1.7, 3.2) | <0.001*** | 2.2 (1.8, 2.6) | <0.001*** |
|  | 4 | 1.1 (0.9, 1.5) | 0.294 | 1.4 (1.3, 1.6) | <0.001*** | 1.0 (0.7, 1.5) | 0.863 | 1.5 (1.2, 1.8) | <0.001*** |
|  | 5 (High) | (Ref) |  | (Ref) |  | (Ref) |  | (Ref) |  |
| **Psycholeptics Medication Use** | No | (Ref) |  | (Ref) |  | (Ref) |  | (Ref) |  |
|  | Yes | 9.0 (7.8, 10.3) | <0.001*** | 7.8 (6.9, 8.8) | <0.001*** | 2.8 (2.2, 3.4) | <0.001*** | 3.0 (2.5, 3.7) | <0.001*** |
| **Psychoanaleptics Medication Use** | No | (Ref) |  | (Ref) |  | (Ref) |  | (Ref) |  |
|  | Yes | 7.3 (6.5, 8.1) | <0.001*** | 6.3 (5.8, 6.8) | <0.001*** | 3.0 (2.5, 3.6) | <0.001*** | 3.3 (2.9, 3.7) | <0.001*** |
| **Comorbid Physical Conditions (Current and Long-Term)** | No Comorbidity | (Ref) |  | (Ref) |  | (Ref) |  | (Ref) |  |
|  | Only 1 | 2.0 (1.5, 2.7) | <0.001*** | 1.6 (1.4, 1.8) | <0.001*** | 2.0 (1.2, 3.3) | 0.009** | 1.9 (1.5, 2.4) | <0.001*** |
|  | 2 - 4 | 4.7 (3.8, 6.0) | <0.001*** | 3.0 (2.7, 3.3) | <0.001*** | 4.3 (2.8, 6.6) | <0.001*** | 3.2 (2.6, 3.9) | <0.001*** |
|  | 5+ | 26.2 (21.0, 32.7) | <0.001*** | 11.6 (10.5, 12.9) | <0.001*** | 11.1 (7.2, 17.1) | <0.001*** | 8.2 (6.6, 10.1) | <0.001*** |
| **Consulted a GP for Health^a^** | No | (Ref) |  | (Ref) |  | (Ref) |  | (Ref) |  |
|  | Yes | 3.5 (2.6, 4.7) | <0.001*** | 2.4 (2.1, 2.8) | <0.001*** | 1.3 (0.8, 2.1) | 0.271 | 1.0 (0.8, 1.3) | 0.942 |
| **Consulted a Psychologist for Health^a^** | No | (Ref) |  | (Ref) |  | (Ref) |  | (Ref) |  |
|  | Yes | 8.1 (7.0, 9.5) | <0.001*** | 7.3 (6.4, 8.2) | <0.001*** | 2.3 (1.8, 2.8) | <0.001*** | 2.6 (2.2, 3.1) | <0.001*** |

***<0.001, **<0.01, and *<0.05. CI, confidential interval; K10, the Kessler Psychological Distress Scale; NA, not available; OR, odds ratio. Model 4 was adjusted for year, sex, age, geographical location, household income, psycholeptics use, psychoanaleptics use, physical comorbidity, whether consulted with a GP, and whether consulted with a psychologist.

a. Data used in Model 4 presented in this table was from the 2011-12 and 2014-15 NHS, since the health service use variables were only available from these two surveys.

**Supplementary Table S3.1.** Odds ratio of mental health-related medication use (psycholeptics and psychoanaleptics) unadjusted and adjusted **(Model 1**: for year, sex, age, geographical location, and household income) for the Australian working age population, 2011-12, 2014-15, and 2017-18

|  |  | **Univariate Analysis** | | | | **Multivariate Analysis (Model 1)** | | | |
| --- | --- | --- | --- | --- | --- | --- | --- | --- | --- |
|  |  | **Psycholeptics Medication Use** | | **Psychoanaleptics Medication Use** | | **Psycholeptics Medication Use** | | **Psychoanaleptics Medication Use** | |
|  |  | **Unadjusted OR (95% CI)** | **p-value** | **Unadjusted OR (95% CI)** | **p-value** | **Adjusted OR (95% CI)** | **p-value** | **Adjusted OR (95% CI)** | **p-value** |
| **Year** | 2011/12 | (Ref) |  | (Ref) |  | (Ref) |  | (Ref) |  |
|  | 2014/15 | 1.0 (0.9, 1.2) | 0.788 | 1.2 (1.1, 1.4) | <0.001*** | 1.0 (0.8, 1.1) | 0.542 | 1.2 (1.1, 1.3) | <0.001*** |
|  | 2017/18 | 1.1 (1, 1.3) | 0.179 | 1.3 (1.2, 1.5) | <0.001*** | 1.0 (0.9, 1.2) | 0.572 | 1.3 (1.1, 1.4) | <0.001*** |
| **Sex** | Male | (Ref) |  | (Ref) |  | (Ref) (, ) |  | (Ref) |  |
|  | Female | 1.0 (0.9, 1.1) | 0.878 | 1.6 (1.4, 1.7) | <0.001*** | 1.0 (0.9, 1.1) | 0.607 | 1.5 (1.4, 1.7) | <0.001*** |
| **Age Group** | 18 - 24 | (Ref) |  | (Ref) |  | (Ref) |  | (Ref) |  |
|  | 25 - 34 | 1.0 (0.7, 1.3) | 0.985 | 1.0 (0.9, 1.2) | 0.744 | 1.1 (0.8, 1.6) | 0.432 | 1.1 (0.9, 1.3) | 0.359 |
|  | 35 - 44 | 1.7 (1.3, 2.2) | <0.001*** | 1.3 (1.1, 1.5) | 0.002** | 1.8 (1.3, 2.4) | 0.001** | 1.3 (1.1, 1.6) | 0.006** |
|  | 45 - 54 | 1.7 (1.3, 2.3) | <0.001*** | 1.6 (1.3, 1.9) | <0.001*** | 1.8 (1.3, 2.5) | <0.001*** | 1.6 (1.3, 1.9) | <0.001*** |
|  | 55 - 64 | 1.8 (1.4, 2.4) | <0.001*** | 1.5 (1.3, 1.8) | <0.001*** | 1.7 (1.2, 2.3) | 0.001** | 1.4 (1.2, 1.7) | <0.001*** |
| **Location** | Major cities | (Ref) |  | (Ref) |  | (Ref) |  | (Ref) |  |
|  | Inner regional | 1.5 (1.3, 1.7) | <0.001*** | 1.4 (1.3, 1.6) | <0.001*** | 1.2 (1.1, 1.4) | 0.004** | 1.3 (1.1, 1.4) | <0.001*** |
|  | Other | 1.0 (0.8, 1.1) | 0.738 | 1.1 (1.0, 1.2) | 0.091 | 0.8 (0.7, 1.0) | 0.022* | 1.0 (0.9, 1.1) | 0.846 |
| **Household Income Quintile** | 1 (Low) | 5.2 (4.3, 6.4) | <0.001*** | 3.2 (2.8, 3.6) | <0.001*** | 5.1 (4.1, 6.2) | <0.001*** | 2.9 (2.6, 3.3) | <0.001*** |
|  | 2 | 4.0 (3.3, 5.0) | <0.001*** | 2.6 (2.3, 3.0) | <0.001*** | 3.9 (3.2, 4.8) | <0.001*** | 2.4 (2.1, 2.8) | <0.001*** |
|  | 3 | 1.4 (1.1, 1.7) | 0.013** | 1.6 (1.4, 1.8) | <0.001*** | 1.3 (1.0, 1.7) | 0.021* | 1.5 (1.3, 1.7) | <0.001*** |
|  | 4 | 1.0 (0.8, 1.3) | 0.706 | 1.2 (1.1, 1.4) | 0.002** | 1.0 (0.8, 1.3) | 0.71 | 1.2 (1.1, 1.4) | 0.005** |
|  | 5 (High) | (Ref) |  | (Ref) |  | (Ref) |  | (Ref) |  |

***<0.001, **<0.01, and *<0.05. CI, confidential interval; OR, odds ratio. Model 1 was adjusted for year, sex, age, geographical location, and household income.

**Supplementary Table S3.2.** Odds ratio of mental health-related medication use (psycholeptics and psychoanaleptics) unadjusted and adjusted (**(Model 2**: for year, sex, age, geographical location, household income, and K10 category) for the Australian working age population, 2011-12, 2014-15, and 2017-18

|  |  | **Univariate Analysis** | | | | **Multivariate Analysis (Model 2)** | | | |
| --- | --- | --- | --- | --- | --- | --- | --- | --- | --- |
|  |  | **Psycholeptics Medication Use** | | **Psychoanaleptics Medication Use** | | **Psycholeptics Medication Use** | | **Psychoanaleptics Medication Use** | |
|  |  | **Unadjusted OR (95% CI)** | **p-value** | **Unadjusted OR (95% CI)** | **p-value** | **Adjusted OR (95% CI)** | **p-value** | **Adjusted OR (95% CI)** | **p-value** |
| **Year** | 2011/12 | (Ref) |  | (Ref) |  | (Ref) |  | (Ref) |  |
|  | 2014/15 | 1.0 (0.9, 1.2) | 0.788 | 1.2 (1.1, 1.4) | <0.001*** | 0.9 (0.8, 1.1) | 0.257 | 1.2 (1.1, 1.3) | 0.001** |
|  | 2017/18 | 1.1 (1, 1.3) | 0.179 | 1.3 (1.2, 1.5) | <0.001*** | 0.9 (0.8, 1.1) | 0.322 | 1.1 (1.0, 1.3) | 0.017* |
| **Sex** | Male | (Ref) |  | (Ref) |  | (Ref) |  | (Ref) |  |
|  | Female | 1.0 (0.9, 1.1) | 0.878 | 1.6 (1.4, 1.7) | <0.001*** | 0.9 (0.8, 1.0) | 0.146 | 1.5 (1.4, 1.6) | <0.001*** |
| **Age Group** | 18 - 24 | (Ref) |  | (Ref) |  | (Ref) |  | (Ref) |  |
|  | 25 - 34 | 1.0 (0.7, 1.3) | 0.985 | 1.0 (0.9, 1.2) | 0.744 | 1.5 (1.0, 2.1) | 0.043* | 1.3 (1.1, 1.6) | 0.007** |
|  | 35 - 44 | 1.7 (1.3, 2.2) | <0.001*** | 1.3 (1.1, 1.5) | 0.002** | 2.2 (1.5, 3.1) | <0.001*** | 1.6 (1.3, 1.9) | <0.001*** |
|  | 45 - 54 | 1.7 (1.3, 2.3) | <0.001*** | 1.6 (1.3, 1.9) | <0.001*** | 2.1 (1.5, 3.0) | <0.001*** | 1.9 (1.6, 2.3) | <0.001*** |
|  | 55 - 64 | 1.8 (1.4, 2.4) | <0.001*** | 1.5 (1.3, 1.8) | <0.001*** | 2.4 (1.7, 3.4) | <0.001*** | 2.0 (1.6, 2.4) | <0.001*** |
| **Location** | Major cities | (Ref) |  | (Ref) |  | (Ref) |  | (Ref) |  |
|  | Inner regional | 1.5 (1.3, 1.7) | <0.001*** | 1.4 (1.3, 1.6) | <0.001*** | 1.3 (1.1, 1.5) | 0.004** | 1.3 (1.2, 1.4) | <0.001*** |
|  | Other | 1.0 (0.8, 1.1) | 0.738 | 1.1 (1.0, 1.2) | 0.091 | 0.8 (0.7, 1.0) | 0.057 | 1.0 (0.9, 1.2) | 0.631 |
| **Household Income Quintile** | 1 (Low) | 5.2 (4.3, 6.4) | <0.001*** | 3.2 (2.8, 3.6) | <0.001*** | 2.2 (1.8, 2.8) | <0.001*** | 1.5 (1.3, 1.7) | <0.001*** |
|  | 2 | 4.0 (3.3, 5.0) | <0.001*** | 2.6 (2.3, 3.0) | <0.001*** | 2.1 (1.7, 2.7) | <0.001*** | 1.5 (1.3, 1.7) | <0.001*** |
|  | 3 | 1.4 (1.1, 1.7) | 0.013** | 1.6 (1.4, 1.8) | <0.001*** | 1.0 (0.8, 1.3) | 0.928 | 1.2 (1.0, 1.3) | 0.022* |
|  | 4 | 1.0 (0.8, 1.3) | 0.706 | 1.2 (1.1, 1.4) | 0.002** | 0.9 (0.7, 1.2) | 0.548 | 1.1 (1.0, 1.3) | 0.187 |
|  | 5 (High) | (Ref) |  | (Ref) |  | (Ref) |  | (Ref) |  |
| **K10 Score** | Low | (Ref) |  | (Ref) |  | (Ref) |  | (Ref) |  |
|  | Moderate | 3.4 (2.8, 4.0) | <0.001*** | 3.2 (2.9, 3.5) | <0.001*** | 2.8 (2.3, 3.4) | <0.001*** | 3.1 (2.8, 3.5) | <0.001*** |
|  | High | 8.6 (7.2, 10.2) | <0.001*** | 7.4 (6.6, 8.2) | <0.001*** | 6.7 (5.5, 8.1) | <0.001*** | 6.6 (5.9, 7.5) | <0.001*** |
|  | Very-high | 20.4 (17.2, 24.3) | <0.001*** | 14.5 (12.8, 16.4) | <0.001*** | 13.9 (11.4, 17.0) | <0.001*** | 11.9 (10.3, 13.7) | <0.001*** |

***<0.001, **<0.01, and *<0.05. CI, confidential interval; K10, the Kessler Psychological Distress Scale; OR, odds ratio. Model 2 was adjusted for year, sex, age, geographical location, household income, and K10 category.

**Supplementary Table S3.3.** Odds ratio of mental health-related medication use (psycholeptics and psychoanaleptics) unadjusted and adjusted (**(Model 4**: for year, sex, age, geographical location, household income, K10 category, physical comorbidity, whether consulted a GP, and whether consulted a psychologist) for the Australian working age population, 2011-12, and 2014-15

|  |  | **Univariate Analysis** | | | | **Multivariate Analysis (Model 4)^a^** | | | |
| --- | --- | --- | --- | --- | --- | --- | --- | --- | --- |
|  |  | **Psycholeptics Medication Use** | | **Psychoanaleptics Medication Use** | | **Psycholeptics Medication Use** | | **Psychoanaleptics Medication Use** | |
|  |  | **Unadjusted OR (95% CI)** | **p-value** | **Unadjusted OR (95% CI)** | **p-value** | **Adjusted OR (95% CI)** | **p-value** | **Adjusted OR (95% CI)** | **p-value** |
| **Year** | 2011/12 | (Ref) |  | (Ref) |  | (Ref) |  | (Ref) |  |
|  | 2014/15 | 1.0 (0.9, 1.2) | 0.788 | 1.2 (1.1, 1.4) | <0.001*** | 0.8 (0.7, 1.0) | 0.013* | 1.1 (0.9, 1.2) | 0.382 |
|  | 2017/18 | 1.1 (1, 1.3) | 0.179 | 1.3 (1.2, 1.5) | <0.001*** | NA |  | NA |  |
| **Sex** | Male | (Ref) |  | (Ref) |  | (Ref) |  | (Ref) |  |
|  | Female | 1.0 (0.9, 1.1) | 0.878 | 1.6 (1.4, 1.7) | <0.001*** | 0.8 (0.6, 0.9) | 0.003** | 1.3 (1.2, 1.5) | <0.001*** |
| **Age Group** | 18 - 24 | (Ref) |  | (Ref) |  | (Ref) |  | (Ref) |  |
|  | 25 - 34 | 1.0 (0.7, 1.3) | 0.985 | 1.0 (0.9, 1.2) | 0.744 | 1.2 (0.8, 1.9) | 0.423 | 1.6 (1.2, 2.1) | 0.002** |
|  | 35 - 44 | 1.7 (1.3, 2.2) | <0.001*** | 1.3 (1.1, 1.5) | 0.002** | 1.7 (1.1, 2.5) | 0.02* | 1.7 (1.3, 2.2) | <0.001*** |
|  | 45 - 54 | 1.7 (1.3, 2.3) | <0.001*** | 1.6 (1.3, 1.9) | <0.001*** | 1.5 (1.0, 2.2) | 0.084 | 1.7 (1.3, 2.2) | <0.001*** |
|  | 55 - 64 | 1.8 (1.4, 2.4) | <0.001*** | 1.5 (1.3, 1.8) | <0.001*** | 1.5 (1.0, 2.3) | 0.058 | 1.7 (1.3, 2.3) | <0.001*** |
| **Location** | Major cities | (Ref) |  | (Ref) |  | (Ref) |  | (Ref) |  |
|  | Inner regional | 1.5 (1.3, 1.7) | <0.001*** | 1.4 (1.3, 1.6) | <0.001*** | 1.2 (1.0, 1.4) | 0.134 | 1.2 (1.0, 1.3) | 0.042* |
|  | Other | 1.0 (0.8, 1.1) | 0.738 | 1.1 (1.0, 1.2) | 0.091 | 0.8 (0.6, 1.0) | 0.034* | 1.0 (0.9, 1.2) | 0.896 |
| **Household Income Quintile** | 1 (Low) | 5.2 (4.3, 6.4) | <0.001*** | 3.2 (2.8, 3.6) | <0.001*** | 1.9 (1.4, 2.5) | <0.001*** | 1.4 (1.1, 1.7) | 0.001** |
|  | 2 | 4.0 (3.3, 5.0) | <0.001*** | 2.6 (2.3, 3.0) | <0.001*** | 1.8 (1.4, 2.4) | <0.001*** | 1.6 (1.3, 1.9) | <0.001*** |
|  | 3 | 1.4 (1.1, 1.7) | 0.013** | 1.6 (1.4, 1.8) | <0.001*** | 1.0 (0.7, 1.4) | 0.955 | 1.3 (1.1, 1.5) | 0.01* |
|  | 4 | 1.0 (0.8, 1.3) | 0.706 | 1.2 (1.1, 1.4) | 0.002** | 0.9 (0.6, 1.2) | 0.426 | 1.1 (0.9, 1.3) | 0.213 |
|  | 5 (High) | (Ref) |  | (Ref) |  | (Ref) |  | (Ref) |  |
| **K10 Score** | Low | (Ref) |  | (Ref) |  | (Ref) |  | (Ref) |  |
|  | Moderate | 3.4 (2.8, 4.0) | <0.001*** | 3.2 (2.9, 3.5) | <0.001*** | 2.1 (1.6, 2.6) | <0.001*** | 2.4 (2.1, 2.8) | <0.001*** |
|  | High | 8.6 (7.2, 10.2) | <0.001*** | 7.4 (6.6, 8.2) | <0.001*** | 4.4 (3.4, 5.6) | <0.001*** | 4.5 (3.8, 5.3) | <0.001*** |
|  | Very-high | 20.4 (17.2, 24.3) | <0.001*** | 14.5 (12.8, 16.4) | <0.001*** | 7.1 (5.4, 9.2) | <0.001*** | 6.5 (5.4, 7.9) | <0.001*** |
| **Comorbid Physical Conditions (Current and Long-Term)** | No comorbidity | (Ref) |  | (Ref) |  | (Ref) |  | (Ref) |  |
|  | Only 1 | 1.8 (1.3, 2.5) | 0.001** | 2.7 (2.2, 3.3) | <0.001*** | 1.4 (0.9, 2.3) | 0.132 | 2.0 (1.6, 2.7) | <0.001*** |
|  | 2 - 4 | 4.0 (3.1, 5.3) | <0.001*** | 5.7 (4.8, 6.8) | <0.001*** | 2.7 (1.8, 3.9) | <0.001*** | 3.3 (2.6, 4.2) | <0.001*** |
|  | 5+ | 14.4 (11, 18.8) | <0.001*** | 16.2 (13.6, 19.2) | <0.001*** | 5.0 (3.3, 7.4) | <0.001*** | 5.0 (3.9, 6.4) | <0.001*** |
| **Consulted a GP for Health in the Last 12 Months^a^** | No | (Ref) |  | (Ref) |  | (Ref) |  | (Ref) |  |
|  | Yes | 4.7 (3.0, 7.6) | <0.001*** | 8.2 (5.7, 11.8) | <0.001*** | 2.5 (1.4, 4.4) | 0.002** | 5.4 (3.4, 8.5) | <0.001*** |
| **Consulted a Psychologist for Health in the Last 12 Months^a^** | No | (Ref) |  | (Ref) |  | (Ref) |  | (Ref) |  |
|  | Yes | 6.3 (5.3, 7.5) | <0.001*** | 8.2 (7.2, 9.4) | <0.001*** | 2.2 (1.8, 2.8) | <0.001*** | 3.6 (3.1, 4.3) | <0.001*** |

***<0.001, **<0.01, and *<0.05. CI, confidential interval; K10, the Kessler Psychological Distress Scale; NA, not available; OR, odds ratio. Model 4 was adjusted for year, sex, age, geographical location, household income, K10 category, physical comorbidity, whether consulted a GP, and whether consulted a psychologist.

a. Data used in Model 4 presented in this table was from the 2011-12 and 2014-15 NHS, since the health service use variables were only available from these two surveys.

**Supplementary Table S4.** Univariate odds ratio of psychological distress and medication use (psycholeptics and psychoanaleptics) for the Australian working age population, 2011/12, 2014/15, and 2017/18

|  |  | **Very-high Psychological Distress** | | **High/Very-high Psychological Distress** | | **Psycholeptics Medication Use** | | **Psychoanaleptics Medication Use** | |
| --- | --- | --- | --- | --- | --- | --- | --- | --- | --- |
|  |  | **Unadjusted OR (95% CI)** | **p-value** | **Unadjusted OR (95% CI)** | **p-value** | **Unadjusted OR (95% CI)** | **p-value** | **Unadjusted OR (95% CI)** | **p-value** |
| **Year** | 2011/12 | (Ref) |  | (Ref) |  | (Ref) |  | (Ref) |  |
|  | 2014/15 | 1.1 (1.0, 1.3) | 0.044* | 1.1 (1.0, 1.2) | 0.003** | 1.0 (0.9, 1.2) | 0.788 | 1.2 (1.1, 1.4) | <0.001*** |
|  | 2017/18 | 1.4 (1.2, 1.5) | <0.001*** | 1.4 (1.3, 1.5) | <0.001*** | 1.1 (1, 1.3) | 0.179 | 1.3 (1.2, 1.5) | <0.001*** |
| **Sex** | Male | (Ref) |  | (Ref) |  | (Ref) |  | (Ref) |  |
|  | Female | 1.5 (1.3, 1.6) | <0.001*** | 1.5 (1.4, 1.6) | <0.001*** | 1.0 (0.9, 1.1) | 0.878 | 1.6 (1.4, 1.7) | <0.001*** |
| **Age Group** | 18 - 24 | (Ref) |  | (Ref) |  | (Ref) |  | (Ref) |  |
|  | 25 - 34 | 0.7 (0.6, 0.8) | <0.001*** | 0.7 (0.6, 0.8) | <0.001*** | 1.0 (0.7, 1.3) | 0.985 | 1.0 (0.9, 1.2) | 0.744 |
|  | 35 - 44 | 0.8 (0.6, 0.9) | 0.004** | 0.7 (0.6, 0.8) | <0.001*** | 1.7 (1.3, 2.2) | <0.001*** | 1.3 (1.1, 1.5) | 0.002** |
|  | 45 - 54 | 1.1 (1.0, 1.4) | 0.142 | 0.8 (0.7, 0.9) | 0.001** | 1.7 (1.3, 2.3) | <0.001*** | 1.6 (1.3, 1.9) | <0.001*** |
|  | 55 - 64 | 1.0 (0.9, 1.2) | 0.79 | 0.8 (0.7, 0.9) | <0.001*** | 1.8 (1.4, 2.4) | <0.001*** | 1.5 (1.3, 1.8) | <0.001*** |
| **Location** | Major Cities | (Ref) |  | (Ref) |  | (Ref) |  | (Ref) |  |
|  | Inner Regional | 1.3 (1.1, 1.4) | <0.001*** | 1.2 (1.1, 1.3) | <0.001*** | 1.5 (1.3, 1.7) | <0.001*** | 1.4 (1.3, 1.6) | <0.001*** |
|  | Other | 1.0 (0.9, 1.2) | 0.541 | 1.0 (1.0, 1.1) | 0.389 | 1.0 (0.8, 1.1) | 0.738 | 1.1 (1.0, 1.2) | 0.091 |
| **Household Income Quintile** | 1 (Low) | 10.1 (8.3, 12.3) | <0.001*** | 6.4 (5.7, 7.2) | <0.001*** | 5.2 (4.3, 6.4) | <0.001*** | 3.2 (2.8, 3.6) | <0.001*** |
|  | 2 | 5.5 (4.5, 6.8) | <0.001*** | 4.0 (3.5, 4.5) | <0.001*** | 4.0 (3.3, 5.0) | <0.001*** | 2.6 (2.3, 3.0) | <0.001*** |
|  | 3 | 2.2 (1.7, 2.7) | <0.001*** | 2.1 (1.8, 2.3) | <0.001*** | 1.4 (1.1, 1.7) | 0.013** | 1.6 (1.4, 1.8) | <0.001*** |
|  | 4 | 1.1 (0.9, 1.5) | 0.294 | 1.4 (1.3, 1.6) | <0.001*** | 1.0 (0.8, 1.3) | 0.706 | 1.2 (1.1, 1.4) | 0.002** |
|  | 5 (High) | (Ref) |  | (Ref) |  | (Ref) |  | (Ref) |  |
| **K10 Score** | Low | NA |  | NA |  | (Ref) |  | (Ref) |  |
|  | Moderate | NA |  | NA |  | 3.4 (2.8, 4.0) | <0.001*** | 3.2 (2.9, 3.5) | <0.001*** |
|  | High | NA |  | NA |  | 8.6 (7.2, 10.2) | <0.001*** | 7.4 (6.6, 8.2) | <0.001*** |
|  | Very-high | NA |  | NA |  | 20.4 (17.2, 24.3) | <0.001*** | 14.5 (12.8, 16.4) | <0.001*** |
| **Psycholeptics Medication Use in the Last 2 Weeks** | No | (Ref) |  | (Ref) | (Ref) | NA |  | NA |  |
|  | Yes | 9.0 (7.8, 10.3) | <0.001*** | 7.8 (6.9, 8.8) | 9.0 (7.8, 10.3) | NA |  | NA |  |
| **Psychoanaleptics Medication Use in the Last 2 Weeks** | No | (Ref) |  | (Ref) | (Ref) | NA |  | NA |  |
|  | Yes | 7.3 (6.5, 8.1) | <0.001*** | 6.3 (5.8, 6.8) | 7.3 (6.5, 8.1) | NA |  | NA |  |
| **Comorbid Physical Conditions (Current and Long-Term)** | No comorbidity | (Ref) |  | (Ref) | (Ref) | (Ref) |  | (Ref) |  |
|  | Only 1 | 2.0 (1.5, 2.7) | <0.001*** | 1.6 (1.4, 1.8) | 2.0 (1.5, 2.7) | 1.8 (1.3, 2.5) | 0.001** | 2.7 (2.2, 3.3) | <0.001*** |
|  | 2 - 4 | 4.7 (3.8, 6.0) | <0.001*** | 3.0 (2.7, 3.3) | 4.7 (3.8, 6.0) | 4.0 (3.1, 5.3) | <0.001*** | 5.7 (4.8, 6.8) | <0.001*** |
|  | 5+ | 26.2 (21.0, 32.7) | <0.001*** | 11.6 (10.5, 12.9) | 26.2 (21.0, 32.7) | 14.4 (11, 18.8) | <0.001*** | 16.2 (13.6, 19.2) | <0.001*** |
| **Consulted a Psychologist for Health in the Last 12 Months^a^** | No | (Ref) |  | (Ref) | (Ref) | (Ref) |  | (Ref) |  |
|  | Yes | 3.5 (2.6, 4.7) | <0.001*** | 2.4 (2.1, 2.8) | 3.5 (2.6, 4.7) | 4.7 (3.0, 7.6) | <0.001*** | 8.2 (5.7, 11.8) | <0.001*** |
| **Consulted a GP for Health in the Last 12 Months^a^** | No | (Ref) |  | (Ref) | (Ref) | (Ref) |  | (Ref) |  |
|  | Yes | 8.1 (7.0, 9.5) | <0.001*** | 7.3 (6.4, 8.2) | 8.1 (7.0, 9.5) | 6.3 (5.3, 7.5) | <0.001*** | 8.2 (7.2, 9.4) | <0.001*** |

***<0.001, **<0.01, and *<0.05. CI, confidential interval; NA, not available; OR, odds ratio.

a. Data related to health service use presented in this table was only available from the 2011-12 and 2014-15 NHS. The 2017-18 NHS did not include questions about health service use.

Interaction effects:

## **Supplementary Table S5.1.** Likelihood-ratio test for logistic regression model of psychological distress, with household income and mental health-related medication use (psycholeptics and/or psychoanaleptics) in the last 2 weeks as predictors, with interaction effects, for the Australian working age population, 2011-12, 2014-15, and 2017-18

|  | **Very-high Psychological Distress** | | | **High/Very-high Psychological Distress** | |
| --- | --- | --- | --- | --- | --- |
|  | **DF** | **Chi-Square** | **p-value** | **Chi-Square** | **p-value** |
| **Household Income Quintile** | 4 | 1.00e+03 | <0.001*** | 1.50e+03 | <0.001*** |
| **Psycholeptics and/or Psychoanaleptics Medication Use** | 1 | 1.30e+03 | <0.001*** | 2.20e+03 | <0.001*** |
| **Household Income Quintile × Psycholeptics and/or Psychoanaleptics Medication Use** | 4 | 18.32 | 0.0011** | 8.05 | 0.0897* |

***<0.001, **<0.01, and *<0.1.

## **Supplementary Table S5.2.** Logistic regression model of psychological distress with household income and mental health-related medication use (psycholeptics and/or psychoanaleptics) in the last 2 weeks as predictors and with interaction effects, for the Australian working age population, 2011-12, 2014-15, and 2017-18

|  | **Very-high Psychological Distress** | | | | **High/Very-high Psychological Distress** | | | |
| --- | --- | --- | --- | --- | --- | --- | --- | --- |
|  | **Coefficient** | **p-value** | **95%** | **CI** | **Coefficient** | **p-value** | **95%** | **CI** |
| **Intercept** | -4.64 | <0.001*** | -4.92 | -4.35 | -3.00 | <0.001*** | -3.13 | -2.87 |
| **Household Income Quintile** | | |  |  |  |  |  |  |
| 1 (Low) | 2.37 | <0.001*** | 2.05 | 2.69 | 1.82 | <0.001*** | 1.65 | 1.98 |
| 2 | 1.83 | <0.001*** | 1.49 | 2.17 | 1.38 | <0.001*** | 1.21 | 1.55 |
| 3 | 0.95 | <0.001*** | 0.59 | 1.31 | 0.82 | <0.001*** | 0.65 | 0.99 |
| 4 | 0.14 | 0.49 | -0.26 | 0.54 | 0.43 | <0.001*** | 0.25 | 0.60 |
| 5 (High) | (Ref) |  |  |  | (Ref) |  |  |  |
| **Psycholeptics and/or Psychoanaleptics Medication Use** | | | | | |  |  |  |
| Yes | 2.35 | <0.001*** | 1.95 | 2.76 | 1.99 | <0.001*** | 1.76 | 2.22 |
| No | (Ref) |  |  |  | (Ref) |  |  |  |
| **Household Income Quintile × Psycholeptics and/or Psychoanaleptics Medication Use** | | | | | | | | |
| 1 (Low) | -0.73 | 0.002** | -1.18 | -0.27 | -0.38 | 0.009** | -0.66 | -0.10 |
| 2 | -0.73 | 0.002** | -1.20 | -0.26 | -0.33 | 0.026* | -0.62 | -0.04 |
| 3 | -0.34 | 0.194 | -0.84 | 0.17 | -0.35 | 0.024* | -0.65 | -0.05 |
| 4 | -0.13 | 0.656 | -0.69 | 0.44 | -0.23 | 0.146 | -0.54 | 0.08 |
| No **×** 5 (High) | (Ref) |  |  |  | (Ref) |  |  |  |

***<0.001, **<0.01, and *<0.1. CI, confidential interval.

## **Supplementary Table S5.3.** Marginal effects of household income and mental health-related medication use (psycholeptics and/or psychoanaleptics) on very-high psychological distress with interaction effects, for the Australian working age population, 2011-12, 2014-15, and 2017-18

|  | **Margin** | **SE** | **z** | **P>z** | **95%** | **CI** |
| --- | --- | --- | --- | --- | --- | --- |
| **Household Income Quintile** | | |  |  |  |  |
| 1 (Low) | 0.14 | 0.01 | 22.83 | <0.001*** | 0.12 | 0.15 |
| 2 | 0.09 | 0.00 | 17.92 | <0.001*** | 0.08 | 0.10 |
| 3 | 0.05 | 0.00 | 14.22 | <0.001*** | 0.04 | 0.05 |
| 4 | 0.02 | 0.00 | 10.45 | <0.001*** | 0.02 | 0.03 |
| 5 (High) | 0.02 | 0.00 | 10.00 | <0.001*** | 0.02 | 0.03 |
| **Psycholeptics and/or Psychoanaleptics Medication Use** | | | | | |  |
| Yes | 0.03 | 0.00 | 23.21 | <0.001*** | 0.03 | 0.03 |
| No | 0.16 | 0.01 | 26.40 | <0.001*** | 0.15 | 0.18 |
| **Household Income Quintile × Psycholeptics and/or Psychoanaleptics Medication Use** | | | | | | |
| 1 (Low) **×** No | 0.09 | 0.01 | 14.78 | <0.001*** | 0.08 | 0.11 |
| 1 (Low) **×** Yes | 0.35 | 0.02 | 21.59 | <0.001*** | 0.31 | 0.38 |
| 2 **×** No | 0.06 | 0.00 | 11.70 | <0.001*** | 0.05 | 0.07 |
| 2 **×** Yes | 0.23 | 0.02 | 15.07 | <0.001*** | 0.20 | 0.26 |
| 3 **×** No | 0.02 | 0.00 | 9.39 | <0.001*** | 0.02 | 0.03 |
| 3 **×** Yes | 0.16 | 0.01 | 10.68 | <0.001*** | 0.13 | 0.19 |
| 4 **×** No | 0.01 | 0.00 | 7.04 | <0.001*** | 0.01 | 0.01 |
| 4 **×** Yes | 0.09 | 0.01 | 7.86 | <0.001*** | 0.07 | 0.12 |
| 5 (High) **×** No | 0.01 | 0.00 | 6.89 | <0.001*** | 0.01 | 0.01 |
| 5 (High) **×** Yes | 0.09 | 0.01 | 7.57 | <0.001*** | 0.07 | 0.12 |

***<0.001, **<0.01, and *<0.1. CI, confidential interval; SE, standard error.

## **Supplementary Table S5.4.** Likelihood-ratio test for logistic regression model of psychological distress, with household income and consultations with a psychologist in the last 12 months on psychological distress as predictors, with interaction effects, for the Australian working age population, 2011-12, and 2014-15

|  | **Very-high Psychological Distress** | | | **High/Very-high Psychological Distress** | |
| --- | --- | --- | --- | --- | --- |
|  | **DF** | **Chi-Square** | **p-value** | **Chi-Square** | **p-value** |
| **Household Income Quintile** | 4 | 1.00e+03 | <0.001*** | 1.50e+03 | <0.001*** |
| **Consulted a Psychologist for Health in the Last 12 Months^a^** | 1 | 510.50 | <0.001*** | 865.63 | <0.001*** |
| **Household Income Quintile × Consulted a Psychologist for Health in the Last 12 Months^a^** | 4 | 10.08 | 0.0391* | 3.19 | 0.5264 |

***<0.001, **<0.01, and *<0.1. a. Data related to health service use presented in this table was only available from the 2011-12 and 2014-15 NHS. The 2017-18 NHS did not include questions about health service use.

## **Supplementary Table S5.5.** Logistic regression model of psychological distress with household income and consultations with a psychologist in the last 12 months as predictors and with interaction effects, for the Australian working age population, 2011-12, and 2014-15

|  | **Very-high Psychological Distress** | | | | **High/Very-high Psychological Distress** | | | |
| --- | --- | --- | --- | --- | --- | --- | --- | --- |
|  | **Coefficient** | **p-value** | **95%** | **CI** | **Coefficient** | **p-value** | **95%** | **CI** |
| **Intercept** | -4.57 | <0.001*** | -4.84 | -4.29 | -3.01 | <0.001*** | -3.14 | -2.87 |
| **Household Income Quintile** | | |  |  |  |  |  |  |
| 1 (Low) | 2.46 | <0.001*** | 2.15 | 2.76 | 1.87 | <0.001*** | 1.71 | 2.03 |
| 2 | 1.88 | <0.001*** | 1.56 | 2.20 | 1.45 | <0.001*** | 1.28 | 1.61 |
| 3 | 0.92 | <0.001*** | 0.58 | 1.26 | 0.78 | <0.001*** | 0.61 | 0.95 |
| 4 | 0.17 | 0.377 | -0.21 | 0.55 | 0.38 | <0.001*** | 0.20 | 0.55 |
| 5 (High) | (Ref) |  |  |  | (Ref) |  |  |  |
| **Consulted a Psychologist for Health in the Last 12 Months^a^** | | | | | |  |  |  |
| Yes | 2.23 | <0.001*** | 1.68 | 2.77 | 1.78 | <0.001*** | 1.43 | 2.12 |
| No | (Ref) |  |  |  | (Ref) |  |  |  |
| **Household Income Quintile × Consulted a Psychologist for Health in the Last 12 Months^a^** | | | | | | | | |
| 1 (Low) | -0.43 | 0.179 | -1.05 | 0.20 | 0.11 | 0.638 | -0.35 | 0.56 |
| 2 | -0.70 | 0.044* | -1.37 | -0.02 | 0.01 | 0.972 | -0.47 | 0.49 |
| 3 | -0.01 | 0.984 | -0.71 | 0.70 | 0.28 | 0.258 | -0.20 | 0.76 |
| 4 | 0.13 | 0.732 | -0.61 | 0.87 | 0.32 | 0.179 | -0.14 | 0.77 |
| No **×** 5 (High) | (Ref) |  |  |  | (Ref) |  |  |  |

***<0.001, **<0.01, and *<0.1. CI, confidential interval.a. Data related to health service use presented in this table was only available from the 2011-12 and 2014-15 NHS. The 2017-18 NHS did not include questions about health service use.

## **Supplementary Table S5.6.** Marginal effects of household income and consultations with a psychologist in the last 12 months on very-high psychological distress with interaction effects, for the Australian working age population, 2011-12, and 2014-15

|  | **Margin** | **SE** | **z** | **P>z** | **95%** | **CI** |
| --- | --- | --- | --- | --- | --- | --- |
| **Household Income Quintile** | | |  |  |  |  |
| 1 (Low) | 0.12 | 0.01 | 19.76 | <0.001*** | 0.11 | 0.14 |
| 2 | 0.07 | 0.00 | 14.94 | <0.001*** | 0.06 | 0.08 |
| 3 | 0.03 | 0.00 | 11.77 | <0.001*** | 0.03 | 0.04 |
| 4 | 0.02 | 0.00 | 9.21 | <0.001*** | 0.01 | 0.02 |
| 5 (High) | 0.01 | 0.00 | 8.44 | <0.001*** | 0.01 | 0.02 |
| **Consulted a Psychologist for Health in the Last 12 Months** | | | | | |  |
| Yes | 0.03 | 0.00 | 26.00 | <0.001*** | 0.03 | 0.04 |
| No | 0.18 | 0.01 | 15.31 | <0.001*** | 0.16 | 0.21 |
| **Household Income Quintile × Consulted a Psychologist for Health in the Last 12 Months** | | | | | | |
| 1 (Low) **×** No | 0.11 | 0.01 | 17.01 | <0.001*** | 0.10 | 0.12 |
| 1 (Low) **×** Yes | 0.42 | 0.03 | 12.77 | <0.001*** | 0.36 | 0.49 |
| 2 **×** No | 0.06 | 0.00 | 13.32 | <0.001*** | 0.05 | 0.07 |
| 2 **×** Yes | 0.24 | 0.03 | 7.07 | <0.001*** | 0.17 | 0.31 |
| 3 **×** No | 0.03 | 0.00 | 10.08 | <0.001*** | 0.02 | 0.03 |
| 3 **×** Yes | 0.19 | 0.03 | 6.10 | <0.001*** | 0.13 | 0.26 |
| 4 **×** No | 0.01 | 0.00 | 7.60 | <0.001*** | 0.01 | 0.02 |
| 4 **×** Yes | 0.12 | 0.02 | 5.21 | <0.001*** | 0.07 | 0.16 |
| 5 (High) **×** No | 0.01 | 0.00 | 7.11 | <0.001*** | 0.01 | 0.01 |
| 5 (High) **×** Yes | 0.09 | 0.02 | 4.56 | <0.001*** | 0.05 | 0.13 |

***<0.001, **<0.01, and *<0.1. CI, confidential interval; SE, standard error.

## **Supplementary Table S5.7.** Likelihood-ratio test for logistic regression model of psychological distress, with household income and mental health-related medication use (psycholeptics and/or psychoanaleptics) in the last 2 weeks plus consultations with a psychologist in the last 12 months as predictors, with interaction effects, for the Australian working age population, 2011-12, and 2014-15

|  | **Very-high Psychological Distress** | | | **High/Very-high Psychological Distress** | |
| --- | --- | --- | --- | --- | --- |
|  | **DF** | **Chi-Square** | **p-value** | **Chi-Square** | **p-value** |
| **Household Income Quintile** | 4 | 1.00e+03 | <0.001*** | 1.50e+03 | <0.001*** |
| **Psycholeptics/Psychoanaleptics Medication Use and/or Consulted a Psychologist** | 3 | 1.00e+03 | <0.001*** | 1.80e+03 | <0.001*** |
| **Household Income Quintile × Psycholeptics/Psychoanaleptics Medication Use and/or Consulted a Psychologist** | 12 | 20.19 | 0.0635* | 11.90 | 0.4534 |

***<0.001, **<0.01, and *<0.1.

## **Supplementary Table S5.8.** Logistic regression model of psychological distress with household income and mental health-related medication use (psycholeptics and/or psychoanaleptics) in the last 2 weeks plus consultations with a psychologist in the last 12 months as predictors and with interaction effects, for the Australian working age population, 2011-12, and 2014-15

|  | **Very-high Psychological Distress** | | | | **High/Very-high Psychological Distress** | | | |
| --- | --- | --- | --- | --- | --- | --- | --- | --- |
|  | **Estimate** | **p-value** | **95%** | **CI** | **Estimate** | **p-value** | **95%** | **CI** |
| **Intercept** | -4.84 | <0.001*** | -5.23 | -4.45 | -3.26 | <0.001*** | -3.44 | -3.07 |
| **Household Income Quintile** | | | | | | | | |
| 1 (Low) | 2.39 | <0.001*** | 1.95 | 2.84 | 1.85 | <0.001*** | 1.62 | 2.08 |
| 2 | 1.81 | <0.001*** | 1.34 | 2.27 | 1.45 | <0.001*** | 1.22 | 1.69 |
| 3 | 0.84 | 0.001** | 0.34 | 1.34 | 0.84 | <0.001*** | 0.60 | 1.07 |
| 4 | -0.06 | 0.836 | -0.64 | 0.52 | 0.42 | 0.001** | 0.18 | 0.67 |
| 5 (High) | (Ref) |  |  |  | (Ref) |  |  |  |
| **Psycholeptics/Psychoanaleptics Medication Use in the Last 2 Weeks and/or Consulted a Psychologist in the Last 12 Months** | | | | | | | | |
| No Medication + Did Not Consult a Psychologist | (Ref) |  |  |  | (Ref) |  |  |  |
| No Medication + Consulted a Psychologist | 1.34 | 0.03* | 0.13 | 2.56 | 1.50 | <0.001*** | 0.92 | 2.08 |
| Took Medication + Did Not Consult a Psychologist | 2.26 | <0.001*** | 1.65 | 2.87 | 2.15 | <0.001*** | 1.82 | 2.48 |
| Took Medication + Consulted a Psychologist | 3.16 | <0.001*** | 2.44 | 3.87 | 2.53 | <0.001*** | 2.03 | 3.02 |
| **Household Income Quintile × Psycholeptics/Psychoanaleptics Medication Use in the Last 2 Weeks and/or Consulted a Psychologist in the Last 12 Months** | | | | | | | | |
| 1 (Low) **×** No Medication + Consulted a Psychologist | 0.57 | 0.404 | -0.76 | 1.90 | 0.25 | 0.526 | -0.52 | 1.02 |
| 1 (Low) **×** Took Medication + Did Not Consult a Psychologist | -0.66 | 0.056* | -1.33 | 0.02 | -0.56 | 0.007** | -0.97 | -0.16 |
| 1 (Low) **×** Took Medication + Consulted a Psychologist | -0.84 | 0.041* | -1.66 | -0.03 | -0.03 | 0.924 | -0.68 | 0.61 |
| 2 **×** No Medication + Consulted a Psychologist | 0.04 | 0.958 | -1.39 | 1.47 | 0.38 | 0.352 | -0.42 | 1.17 |
| 2 **×** Took Medication + Did Not Consult a Psychologist | -0.63 | 0.08* | -1.33 | 0.07 | -0.52 | 0.014* | -0.94 | -0.11 |
| 2 **×** Took Medication + Consulted a Psychologist | -0.87 | 0.053* | -1.74 | 0.01 | -0.08 | 0.815 | -0.76 | 0.60 |
| 3 **×** No Medication + Consulted a Psychologist | 0.76 | 0.301 | -0.68 | 2.19 | 0.36 | 0.367 | -0.42 | 1.13 |
| 3 **×** Took Medication + Did Not Consult a Psychologist | -0.10 | 0.792 | -0.85 | 0.65 | -0.41 | 0.064* | -0.84 | 0.02 |
| 3 **×** Took Medication + Consulted a Psychologist | -0.09 | 0.842 | -1.03 | 0.84 | 0.09 | 0.799 | -0.61 | 0.79 |
| 4 **×** No Medication + Consulted a Psychologist | 0.79 | 0.328 | -0.79 | 2.36 | 0.40 | 0.304 | -0.36 | 1.16 |
| 4 **×** Took Medication + Did Not Consult a Psychologist | 0.14 | 0.756 | -0.73 | 1.01 | -0.33 | 0.154 | -0.78 | 0.12 |
| 4 **×** Took Medication + Consulted a Psychologist | 0.17 | 0.731 | -0.82 | 1.16 | 0.24 | 0.476 | -0.42 | 0.90 |
| 5 (High) **×** No Medication + Did Not Consult a Psychologist | (Ref) |  |  |  | (Ref) |  |  |  |

***<0.001, **<0.01, and *<0.1. CI, confidential interval.

## **Supplementary Table S5.9.** Marginal effects of household income and mental health-related medication use (psycholeptics and/or psychoanaleptics) in the last 2 weeks plus consultations with a psychologist in the last 12 months on very-high psychological distress with interaction effects, for the Australian working age population, 2011-12, and 2014-15

|  | **Margin** | **SE** | **z** | **P>z** | **95%** | **CI** |
| --- | --- | --- | --- | --- | --- | --- |
| **Household Income Quintile** | | |  |  |  |  |
| 1 (Low) | 0.13 | 0.01 | 18.15 | <0.001*** | 0.11 | 0.14 |
| 2 | 0.08 | 0.01 | 13.80 | <0.001*** | 0.07 | 0.09 |
| 3 | 0.04 | 0.00 | 11.48 | <0.001*** | 0.04 | 0.05 |
| 4 | 0.02 | 0.00 | 8.17 | <0.001*** | 0.02 | 0.03 |
| 5 (High) | 0.02 | 0.00 | 7.82 | <0.001*** | 0.02 | 0.03 |
| **Psycholeptics/Psychoanaleptics Medication Use in the Last 2 Weeks and Consulted a Psychologist in the Last 12 Months** | | | | | | |
| No Medication + Did Not Consult a Psychologist | 0.03 | 0.00 | 16.43 | <0.001*** | 0.02 | 0.03 |
| No Medication + Consulted a Psychologist | 0.12 | 0.02 | 7.87 | <0.001*** | 0.09 | 0.16 |
| Took Medication + Did Not Consult a Psychologist | 0.14 | 0.01 | 16.74 | <0.001*** | 0.12 | 0.15 |
| Took Medication + Consulted a Psychologist | 0.25 | 0.02 | 12.49 | <0.001*** | 0.21 | 0.29 |
| **Household Income Quintile & Took Medication in the Last 2 Weeks or Consulted a Psychologist in the Last 12 Months** | | | | | | |
| 1 (Low) **×** No Medication + Did Not Consult a Psychologist | 0.08 | 0.01 | 10.63 | <0.001*** | 0.07 | 0.09 |
| 1 (Low) **×** No Medication + Consulted a Psychologist | 0.37 | 0.06 | 6.17 | <0.001*** | 0.25 | 0.49 |
| 1 (Low) **×** Took Medication + Did Not Consult a Psychologist | 0.30 | 0.02 | 13.42 | <0.001*** | 0.26 | 0.34 |
| 1 (Low) **×** Took Medication + Consulted a Psychologist | 0.47 | 0.04 | 10.87 | <0.001*** | 0.38 | 0.55 |
| 2 **×** No Medication + Did Not Consult a Psychologist | 0.05 | 0.01 | 8.32 | <0.001*** | 0.04 | 0.06 |
| 2 **×** No Medication + Consulted a Psychologist | 0.16 | 0.05 | 3.27 | 0.001** | 0.06 | 0.26 |
| 2 **×** Took Medication + Did Not Consult a Psychologist | 0.20 | 0.02 | 9.73 | <0.001*** | 0.16 | 0.24 |
| 2 **×** Took Medication + Consulted a Psychologist | 0.32 | 0.05 | 6.43 | <0.001*** | 0.22 | 0.42 |
| 3 **×** No Medication + Did Not Consult a Psychologist | 0.02 | 0.00 | 6.54 | <0.001*** | 0.01 | 0.02 |
| 3 **×** No Medication + Consulted a Psychologist | 0.13 | 0.04 | 3.22 | 0.001** | 0.05 | 0.21 |
| 3 **×** Took Medication + Did Not Consult a Psychologist | 0.14 | 0.02 | 7.14 | <0.001*** | 0.10 | 0.17 |
| 3 **×** Took Medication + Consulted a Psychologist | 0.28 | 0.05 | 5.28 | <0.001*** | 0.18 | 0.39 |
| 4 **×** No Medication + Did Not Consult a Psychologist | 0.01 | 0.00 | 4.60 | <0.001*** | 0.00 | 0.01 |
| 4 **×** No Medication + Consulted a Psychologist | 0.06 | 0.03 | 2.30 | 0.021** | 0.01 | 0.11 |
| 4 **×** Took Medication + Did Not Consult a Psychologist | 0.08 | 0.02 | 4.77 | <0.001*** | 0.04 | 0.11 |
| 4 **×** Took Medication + Consulted a Psychologist | 0.17 | 0.04 | 4.40 | <0.001*** | 0.10 | 0.25 |
| 5 (High) **×** No Medication + Did Not Consult a Psychologist | 0.01 | 0.00 | 5.02 | <0.001*** | 0.00 | 0.01 |
| 5 (High) **×** No Medication + Consulted a Psychologist | 0.03 | 0.02 | 1.76 | 0.079* | 0.00 | 0.06 |
| 5 (High) **×** Took Medication + Did Not Consult a Psychologist | 0.07 | 0.02 | 4.52 | <0.001*** | 0.04 | 0.10 |
| 5 (High) **×** Took Medication + Consulted a Psychologist | 0.16 | 0.04 | 3.93 | <0.001*** | 0.08 | 0.23 |

***<0.001, **<0.01, and *<0.1. CI, confidential interval; SE, standard error.

## **Supplementary Figure S5.10.** Adjusted predictions of household income and mental health-related medication use (psycholeptics and/or psychoanaleptics) in the last 2 weeks plus consultations with a psychologist in the last 12 months on the probability for very-high psychological distress, among the Australian working age population, 2011-12, and 2014-15^$, %^

**
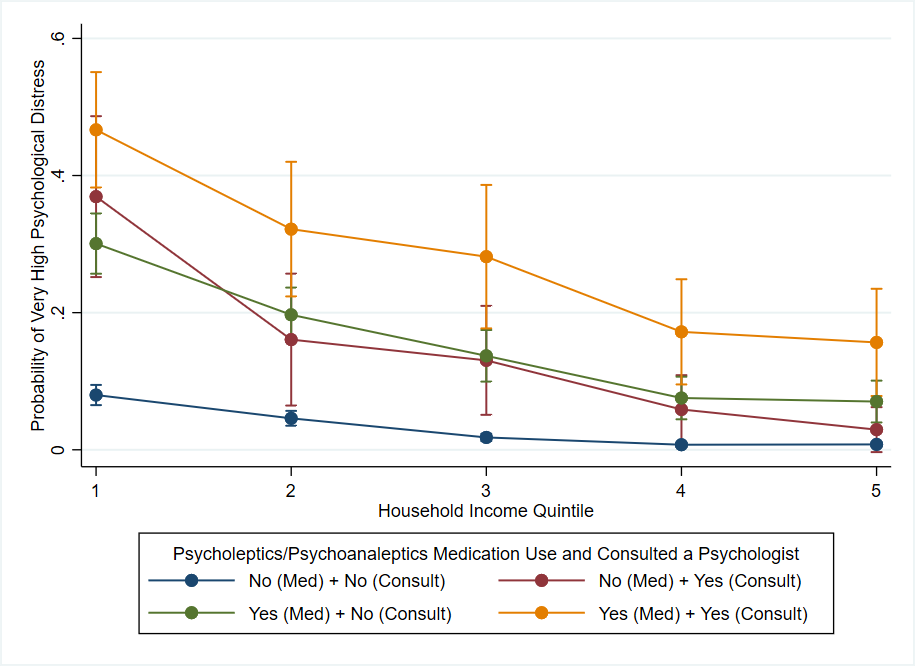
**

$. This figure only included working-aged participants from the 2011-12 and 2014-15 NHS, because data related to health service use presented in this figure was only available from the 2011-12 and 2014-15 NHS. The 2017-18 NHS did not include questions about health service use.

%. The interaction effect was statistically significant (p = .0635), using a threshold of 0.1 (Sun et al., 2010), between household income and mental health-related medication use (psycholeptics and/or psychoanaleptics) in the last 2 weeks plus consultations with a psychologist in the last 12 months on the prevalence of very-high psychological distress.

**Supplementary Table S6.** Number of participants in each variable category^†^

|  |  | **Total** | **Psychological distress** | **Psycholeptics and/or Psychoanaleptics Medication Use** | **Consulted a Psychologist for Health^b^** |
| --- | --- | --- | --- | --- | --- |
| **Year** | 2011 - 12 | 12,332 | 12,281 | 8,550 | 12,332 |
|  | 2014 - 15 | 11,296 | 11,235 | 7,946 | 11,296 |
|  | 2017 - 18 | 12,183 | 11,794 | 8,230 | NA |
| **Age Group** | 18 - 24 | 3,525 | 3,443 | 1,929 | 2,351 |
|  | 25 - 34 | 7,776 | 7,668 | 4,926 | 5,200 |
|  | 35 - 44 | 8,582 | 8,480 | 5,665 | 5,738 |
|  | 45 - 54 | 8,179 | 8,061 | 5,847 | 5,379 |
|  | 55 - 64 | 7,749 | 7,658 | 6,359 | 4,960 |
| **Sex** | Male | 16,782 | 16,478 | 10,489 | 11,068 |
|  | Female | 19,029 | 18,832 | 14,237 | 12,560 |
| **Household Income Quintile** | 1 (Low) | 4,167 | 4,103 | 3,026 | 2,628 |
|  | 2 | 4,315 | 4,231 | 3,068 | 2,774 |
|  | 3 | 6,219 | 6,137 | 4,177 | 4,055 |
|  | 4 | 7,460 | 7,387 | 5,079 | 4,884 |
|  | 5 (High) | 7,740 | 7,660 | 5,514 | 5,076 |
| **Psycholeptics and/or Psychoanaleptics Medication Use** **in the Last 2 Weeks** | Yes | 3,969 | 3,890 | NA | 2,522 |
|  | No | 20,757 | 20,536 | NA | 13,974 |
| **Psycholeptics and/or Psychoanaleptics Medication Use by Household Income Quintile** | 1 (Low) | 896 | 883 | NA | 561 |
|  | 2 | 763 | 743 | NA | 484 |
|  | 3 | 618 | 606 | NA | 393 |
|  | 4 | 607 | 598 | NA | 374 |
|  | 5 (High) | 567 | 562 | NA | 355 |
| **Did Not Took Psycholeptics or Psychoanaleptics Medication by Household Income Quintile** | 1 (Low) | 2,130 | 2,107 | NA | 1,374 |
|  | 2 | 2,305 | 2,267 | NA | 1,505 |
|  | 3 | 3,559 | 3,524 | NA | 2,397 |
|  | 4 | 4,472 | 4,440 | NA | 2,931 |
|  | 5 (High) | 4,947 | 4,900 | NA | 3,288 |
| **Consulted a Psychologist for Health in the Last 12 Months^a^** | Yes | 1,165 | 1,151 | 1,021 | NA |
|  | No | 22,463 | 22,365 | 15,475 | NA |
| **Consulted a Psychologist^a^ by Household Income Quintile** | 1 (Low) | 225 | 222 | 202 | NA |
|  | 2 | 162 | 158 | 147 | NA |
|  | 3 | 156 | 155 | 140 | NA |
|  | 4 | 210 | 208 | 180 | NA |
|  | 5 (High) | 218 | 216 | 187 | NA |
| **Did Not Consult a Psychologist^a^ by Household Income Quintile** | 1 (Low) | 2,403 | 2,383 | 1,733 | NA |
|  | 2 | 2,612 | 2,590 | 1,842 | NA |
|  | 3 | 3,899 | 3,895 | 2,650 | NA |
|  | 4 | 4,674 | 4,668 | 3,125 | NA |
|  | 5 (High) | 4,858 | 4,855 | 3,456 | NA |
| **Both Took Psycholeptics and/or Psychoanaleptics Medications and Consulted a Psychologist^a^** | Yes | 568 | 557 | NA | NA |
|  | No | 15,928 | 15,858 | NA | NA |
| **Both Took Psycholeptics and/or Psychoanaleptics Medications and Consulted a Psychologist^a^ by Household Income Quintile** | 1 (Low) | 137 | 135 | NA | NA |
|  | 2 | 91 | 87 | NA | NA |
|  | 3 | 71 | 71 | NA | NA |
|  | 4 | 94 | 93 | NA | NA |
|  | 5 (High) | 85 | 83 | NA | NA |
| **Did Not Take Psycholeptics and/or Psychoanaleptics Medications nor Consulted a Psychologist^a^ by Household Income Quintile** | 1 (Low) | 1,309 | 1,302 | NA | NA |
|  | 2 | 1,449 | 1,437 | NA | NA |
|  | 3 | 2,328 | 2,327 | NA | NA |
|  | 4 | 2,845 | 2,842 | NA | NA |
|  | 5 (High) | 3,186 | 3,183 | NA | NA |
| **Consulted a GP for Health in the Last 12 Months^a^** | Yes | 20,030 | 19,932 | 14,938 | 20,030 |
|  | No | 3,589 | 3,575 | 1,554 | 3,589 |
| **Consulted a GP^a^ by Household Income Quintile** | 1 (Low) | 2,259 | 2,238 | 1,788 | 2,259 |
|  | 2 | 2,382 | 2,357 | 1,837 | 2,382 |
|  | 3 | 3,412 | 3,407 | 2,500 | 3,412 |
|  | 4 | 4,153 | 4,145 | 2,992 | 4,153 |
|  | 5 (High) | 4,351 | 4,346 | 3,291 | 4,351 |
| **Did Not Consult a GP^a^ by Household Income Quintile** | 1 (Low) | 365 | 363 | 147 | 365 |
|  | 2 | 391 | 390 | 152 | 391 |
|  | 3 | 642 | 642 | 289 | 642 |
|  | 4 | 730 | 730 | 312 | 730 |
|  | 5 (High) | 724 | 724 | 351 | 724 |
| **Time Since Last Consulted GP for Own Health^a^** | Never/Less than 3 months ago | 12,373 | 12,296 | 9,910 | 12,320 |
|  | 3 - 6 months | 4,731 | 4,718 | 3,288 | 4,731 |
|  | 6 - 9 months | 1,914 | 1,908 | 1,154 | 1,914 |
|  | 9 - 12 months | 1,000 | 998 | 558 | 1,000 |
|  | 1 - 2 years | 1,954 | 1,950 | 919 | 1,954 |
|  | More than 2 years | 1,545 | 1,537 | 608 | 1,545 |
| **Psycholeptics Medication Use** **in the Last 2 Weeks** | Yes | 1,239 | 1,202 | NA | 804 |
|  | No | 23,487 | 23,224 | NA | 15,692 |
| **Psychoanaleptics Medication Use** **in the Last 2 Weeks** | Yes | 3,330 | 3,273 | NA | 2,099 |
|  | No | 21,396 | 21,153 | NA | 14,397 |
| **K10 Category** | Low | 23,256 | NA | 15,174 | 15,990 |
|  | Moderate | 7,350 | NA | 5,428 | 4,635 |
|  | High | 3,077 | NA | 2,428 | 1,895 |
|  | Very-high | 1,627 | NA | 1,396 | 996 |
| **Location** | Major cities | 23,174 | 22,857 | 16,124 | 15,603 |
|  | Inner regional | 6,445 | 6,363 | 4,533 | 4,132 |
|  | Other | 6,192 | 6,090 | 4,069 | 3,893 |
| **Australian Born** | Yes | 25,635 | 25,295 | 17,977 | 17,038 |
|  | No | 10,176 | 10,015 | 6,749 | 6,590 |
| **Completed Tertiary Education** | Yes | 23,594 | 23,363 | 16,512 | 15,646 |
|  | No | 11,473 | 11,210 | 7,691 | 7,632 |
| **Employment Status** | Employed | 27,592 | 27,264 | 18,591 | 18,227 |
|  | Unemployed | 1,209 | 1,189 | 775 | 801 |
|  | Not in the Labour Force | 7,010 | 6,857 | 5,360 | 4,600 |
| **Hours Working/Week** | Not in the Labour Force | 7,010 | 6,857 | 5,360 | 4,600 |
|  | 24 hours or less | 5,029 | 4,964 | 3,593 | 3,294 |
|  | 25 - 39 hours | 9,367 | 9,282 | 6,409 | 6,098 |
|  | 40 - 49 hours | 8,010 | 7,905 | 5,238 | 5,327 |
|  | 50 hours or more | 5,186 | 5,113 | 3,351 | 3,508 |
| **Feeling Depressed (Current and Long-Term)^b^** | Yes | 2,295 | 1,553 | 1,292 | 1,419 |
|  | No | 33,516 | 21,476 | 14,884 | 22,209 |
| **Depression (Current and Long-Term)^b^** | Yes | 3,458 | 2,203 | 2,025 | 2,188 |
|  | No | 32,353 | 20,826 | 14,151 | 21,440 |
| **Anxiety Disorders (Current and Long-Term)^b^** | Yes | 1,712 | 1,658 | 1,494 | 687 |
|  | No | 21,767 | 21,371 | 14,682 | 10,609 |
| **Mood Disorders (Current and Long-Term)^b, c^** | Yes | 3,407 | 3,318 | 2,926 | 1,534 |
|  | No | 20,072 | 19,711 | 13,250 | 9,762 |
| **Anxiety Related Problems (Current and Long-Term)^b, d^** | Yes | 4,231 | 3,548 | 3,038 | 2,211 |
|  | No | 31,580 | 19,481 | 13,138 | 21,417 |
| **Mood and Anxiety Related Disorders (Current and Long-Term)^b, e^** | Yes | 5,043 | 4,923 | 4,249 | 2,273 |
|  | No | 18,436 | 18,106 | 11,927 | 9,023 |
| **Comorbid Physical Conditions (Current and Long-Term)** | No comorbidity | 11,406 | 11,225 | 5,737 | 7,552 |
|  | Only 1 | 6,683 | 6,590 | 4,305 | 4,528 |
|  | 2 - 4 | 11,696 | 11,559 | 9,207 | 7,774 |
|  | 5+ | 6,026 | 5,936 | 5,477 | 3,774 |

NA, not available. †. All cell sizes had >50 observations

a. Data related to health service use presented in this table was only available from the 2011-12 and 2014-15 NHS. The 2017-18 NHS did not include questions about health service use.

b. Data related to the mental health conditions presented in this table was only from the 2014-15 and 2017-18 NHS, since these mental health conditions were directly comparable between the 2014-15 and 2017-18 NHS but not comparable between 2011-12 and 2014-15 NHS.

c. Conditions recorded in the “Mood Disorders” variable included “feeling depressed”, “other mood (affective) disorders” and “depression”, which were classified based on the 10^th^ Revision of the International Classification of Diseases (ICD10).

d. Conditions recorded in the “Anxiety Related Problems” variable included “feeling anxious, nervous or tense”, “anxiety disorders”, “panic disorders/panic attacks”, “phobic anxiety disorders”, “post-traumatic stress disorder”, and “obsessive compulsive disorder”, which were classified based on the 10^th^ Revision of the International Classification of Diseases (ICD10).

e. Conditions recorded in the “Mood and Anxiety Related Disorders” variable included mood disorders, such as “feeling depressed”, “other mood (affective) disorders” and “depression”, and anxiety related problems, such as “feeling anxious, nervous or tense”, “anxiety disorders”, “panic disorders/panic attacks”, “phobic anxiety disorders”, “post-traumatic stress disorder”, and “obsessive compulsive disorder”. These conditions were classified based on the 10^th^ Revision of the International Classification of Diseases (ICD10).

**Supplementary Table S7.** Comparability of variables between the 2011-12, 2014-15, and 2017-18 National Health Surveys (NHS)^^[[1]](#footnote-1)^^

|  | 2011-12 NHS versus 2014-15 NHS | 2014-15 NHS versus 2017-18 NHS |
| --- | --- | --- |
| Psychological Distress  (Kessler 10 score) | Directly comparable | Directly comparable |
| Mental Health-Related Medication Use^^[[2]](#footnote-2)^^  (Type of medication taken in the last 2 weeks) | Directly comparable | Directly comparable |
|  |  |  |
| Long-Term Mental Health Conditions^^[[3]](#footnote-3)^^  (Self-reported mental health conditions that lasted, or expected to last, for 6 months or more) | Not comparable  (Note: From the 2014-15 NHS onwards, information of mental and behaviour conditions was collected in the new Mental, Behavioural and Cognitive Conditions module, while such information was collected as a part of the Long-Term Conditions module in the previous two surveys. This shift in collection methodology has resulted in a significant increase in the number of people who reported having a mental and behavioural condition in the 2014-15 NHS. This could possibly be explained by the higher prominence of mental health and behavioural conditions due to the introduction of the new module. In addition, more mental health conditions were included in the prompt card O1 in the 2014-15 NHS, resulting in increased response options for participants.) | Directly comparable  (Note: The 2017-18 NHS included an additional example of '32. Learning difficulties, including dyslexia' in the prompt card O2.) |
| Consultations with a psychologist^^[[4]](#footnote-4)^^  (Whether participants have consulted with a psychologist for health in the last 12 months) | Directly comparable  (Note: the Health Service Use module in the 2014-15 NHS was originally named the Healthy Lifestyles module.) | Not comparable  (The 2017-18 NHS did not include questions about health service use) |
| Physical Comorbidity^^[[5]](#footnote-5)^^  (Self-reported current and long-term comorbid physical conditions) | Generally comparable  (Note: Comorbid conditions were based on both self-reported conditions and biomedical data in the 2011-12 NHS, while it is only based on self-reported conditions in the 2014-15 NHS. Several conditions including “stroke”, “back problems (dorsopathies)”, “back pain/problem, disc disorder”, “gout”, and “rheumatism” presented some issues in the 2011-14 NHS and were not relatively comparable to the 2014-15 NHS) | Directly comparable |
| Equivalised Household Income  (Total household income adjusted by the equivalence scale (in deciles)) | Directly comparable | Directly comparable |
| Gender  (Sex of person (female or male)) | Directly comparable | Directly comparable |
| Age  (Age of person (years in categories)) | Directly comparable | Directly comparable |
| Country of Birth  (Whether is Australian born or not) | Directly comparable | Directly comparable |
| Geographical Location^^[[6]](#footnote-6)^^  (Remoteness area categories) | Directly comparable | Directly comparable |
| Highest Educational Attainment  (Whether completed tertiary education or not) | Directly comparable  (Note: The 2011-12 NHS collected the levels of received education based on an interviewer coded list and other text information, while the 2014-15 NHS used a trigram coder.) | Directly comparable |
| Employment Status  (Whether is employed, or unemployed, or not in the labour force) | Directly comparable | Directly comparable |
| Average Working Hours  (Hours usually worked each week (hours in categories)) | Directly comparable | Directly comparable |

**Supplementary Table S8.** Additional data items used in supplementary data analysis

Self-reported current and long-term mental health symptoms, including feeling depressed, feeling anxious, nervous, or tense, depression, anxiety disorders, panic disorders/panic attacks, phobic anxiety disorders, post-traumatic stress disorder, and obsessive-compulsive disorder, which lasted or expected to last for 6 months or more, were used in this study as complementary variables to psychological distress. However, data of mental disorders was only comparable between 2014-15 and 2017-18 NHS, which was included in this study, and was not comparable between 2011-12 and 2014-15 NHS due to a shift in collection methodology from 2014-15 onwards (**Supplementary Table S7).**

Information regarding consultations with a GP was available in the 2011-12 and 2014-15 NHS (but not the 2017-18 NHS) (Supplementary Table S7) by asking participants whether they had consulted a GP for health in the last 12 months.

**Supplementary Material S9.** Details about the results related to time trend and sociodemographic factors

## **Time trend**

The prevalence of very-high and high/very-high psychological distress increased from 3.9% [95% CI: 3.6-4.3] and 11.8% [95% CI: 11.2-12.4] in 2011-12 to 5.4% [95% CI: 5.0-5.8] and 15.6% [95% CI: 14.9-16.3] in 2017-18 (**Table 1**). In regression Model 3, the rates of high/very-high distress in 2017-18 became 1.2 [95% CI: 1.1-1.3] times greater than the rates in 2011-12 (p < .001) (**Table 3**). Additionally, an increase in the percentage of psycholeptics and psychoanaleptics intake from 4.6% [95% CI: 4.1-5.0] and 11.2% [95% CI: 10.5-11.9] to 5.1% [95% CI: 4.6-5.6] and 14.6% [95% CI: 13.8-15.4] was recorded from 2011-12 to 2017-18 (**Table 1**). A statistically significant increase in psychoanaleptics medication use was reported over three consecutive survey years, after adjusting for socioeconomic and psychological distress factors (Model 2; **Supplementary Table 3.2**). The relationship remained significant between 2014-15 and 2017-18 with OR of 1.2 ([95% CI: 1.0-1.3], p = .009), after further adjusting for physical comorbidity (Model 3; **Table 4**). Furthermore, the rates of consulting a psychologist for health also climbed from 3.3% [95% CI: 3.0-3.7] to 6.6% [6.2-7.1] (**Table 1**).

## **Supplementary Table S9.1. Temporal Changes in the Prevalence of Psychological Distress, Use of Mental Health-Related Medications (Psycholeptics and/or Psychoanaleptics), and Consultations with Psychologists Among the Australian Working-Age Population, in 2014-15 and 2017-18 compared to 2011-12**

|  |  |  | **Very-high psychological distress** | | **High/very-high psychological distress** | | **Psycholeptics and/or Psychoanaleptics Medication Use** | | **Consulted a Psychologist for Health in the Last 12 Months^§^** | |
| --- | --- | --- | --- | --- | --- | --- | --- | --- | --- | --- |
| **Survey Year and Household Income Quintile** | **Total %** | **∆Rate Difference%** | **Rate% (95% CI)** | **∆Rate Difference%** | **Rate% (95% CI)** | **∆Rate Difference%** | **Rate% (95% CI)** | **∆Rate Difference%** | **Rate% (95% CI)** | **∆Rate Difference%** |
| 2011-12 and 1 (Low) | 12.5 | - | 12.59 (10.81, 14.54) | - | 27.62 (25.17, 30.16) | - | 28.26 (25.41, 31.23) | - | 6.14 (4.89, 7.60) | - |
| 2014-15 and 1 (Low) | 14.7 | 2.13 | 14.39 (12.55, 16.39) | 1.80 | 28.49 (26.08, 30.99) | 0.87 | 29.70 (26.86, 32.67) | 1.44 | 10.88 (9.26, 12.67) | 4.74 |
| 2017-18 and 1 (Low) | 14.7 | 2.15 | 14.69 (12.93, 16.58) | 2.10 | 32.71 (30.34, 35.15) | 5.09 | 30.71 (27.98, 33.54) | 2.45 | NA | NA |
| 2011-12 and 2 | 14.4 | - | 7.77 (6.45, 9.25) | - | 18.87 (16.90, 20.97) | - | 23.20 (20.66, 25.88) | - | 4.47 (3.47, 5.65) | - |
| 2014-15 and 2 | 14.2 | -0.22 | 7.03 (5.69, 8.57) | -0.74 | 20.39 (18.21, 22.70) | 1.52 | 25.58 (22.83, 28.48) | 2.38 | 7.40 (6.04, 8.96) | 2.93 |
| 2017-18 and 2 | 14.7 | 0.31 | 9.51 (8.06, 11.12) | 1.74 | 23.26 (21.13, 25.50) | 4.39 | 25.86 (23.27, 28.58) | 2.66 | NA | NA |
| 2011-12 and 3 | 21.2 | - | 3.22 (2.52, 4.05) | - | 10.45 (9.20, 11.81) | - | 13.41 (11.71, 15.26) | - | 2.11 (1.55, 2.81) | - |
| 2014-15 and 3 | 20.5 | -0.65 | 3.14 (2.40, 4.03) | -0.08 | 11.93 (10.50, 13.48) | 1.48 | 14.84 (12.96, 16.87) | 1.43 | 5.85 (4.83, 7.01) | 3.74 |
| 2017-18 and 3 | 20.6 | -0.55 | 3.79 (3.01, 4.70) | 0.57 | 13.61 (12.16, 15.15) | 3.16 | 16.22 (14.32, 18.27) | 2.81 | NA | NA |
| 2011-12 and 4 | 25.3 | - | 1.58 (1.14, 2.14) | - | 7.52 (6.53, 8.60) | - | 10.20 (8.82, 11.72) | - | 2.77 (2.18, 3.48) | - |
| 2014-15 and 4 | 25.0 | -0.28 | 1.75 (1.25, 2.38) | 0.17 | 8.63 (7.51, 9.86) | 1.11 | 12.55 (10.95, 14.29) | 2.35 | 6.03 (5.09, 7.08) | 3.26 |
| 2017-18 and 4 | 24.6 | -0.72 | 2.07 (1.55, 2.71) | 0.49 | 9.72 (8.59, 10.94) | 2.20 | 13.13 (11.60, 14.80) | 2.93 | NA | NA |
| 2011-12 and 5 (High) | 26.6 | - | 1.21 (0.83, 1.69) | - | 5.24 (4.44, 6.15) | - | 9.09 (7.85, 10.46) | - | 3.22 (2.59, 3.96) | - |
| 2014-15 and 5 (High) | 25.6 | -0.97 | 1.54 (1.08, 2.12) | 0.33 | 5.76 (4.85, 6.78) | 0.52 | 10.50 (9.08, 12.05) | 1.41 | 5.54 (4.65, 6.55) | 2.32 |
| 2017-18 and 5 (High) | 25.4 | -1.19 | 5.35 (4.31, 6.56) | 4.14 | 7.65 (6.65, 8.74) | 2.41 | 11.33 (9.93, 12.85) | 2.24 | NA | NA |

CI, confidence interval; NA, not available $. All cell sizes had >50 observations. §. Data related to health service use presented in this table was only available from 2011-12 and 2014-15 NHS. The 2017-18 NHS did not include questions about health service use.

## **Sociodemographic factors**

According to the descriptive analysis (**Table 1 and Supplementary Table 1**), higher rates of psychological distress and mental health-related medication use were linked with various factors, including lower income, living in inner regional areas, being female, not completing tertiary education, being unemployed or not in the labour force, fewer working hours, use of psycholeptics or psychoanaleptics (distress rates only), higher levels of psychological distress (medication use rates only), having mood or anxiety-related disorders, consultations with a GP or psychologist in the last 12 months, shorter time period since the last GP visit, and multiple physical comorbidities.

Regression (Model 3; **Table 3**) identified a significant and positive relationship for high/very-high psychological distress with being younger, living in major cities, having lower income, using medications, and having greater physical comorbidities (p < .01). In addition, both psycholeptics and psychoanaleptics intake were positively connected with being older, living in inner regional areas, having lower income, having higher psychological distress levels, and having greater physical comorbidities (p < .05) (Model 3; **Table 4**).

**Supplementary Material S10.** Treatment Prevalence Paradox (TPP)

Considering the pressing need to explain the Treatment Prevalence Paradox (TPP), this study attempted to shed light on the dynamics among income levels, psychological distress, the use of mental health medications, and the utilization of psychologist services in Australia, so that policy and service reforms can target people with the greatest needs. This study explored the TPP in relation to the noticeable increases in mental health treatment rates and provision of care after the introduction of the Better Access Scheme in 2006 (DoHAC, 2023), which has not led to decrease in the prevalence of mental health conditions. The Better Access Scheme expanded Medicare Benefits Schedule (MBS) items to support people with mental health disorders under Australia’s universal health insurance scheme (DoHAC, 2023). As a result, increased treatment rates were observed over the years (Looi et al., 2022; Pirkis et al., 2022; Whiteford et al., 2014), together with the massive growth in mental health expenditure at an average annual rate of 4% (AIHW, 2020-21; Pirkis et al., 2022). However, there has been no clear evidence of national improvement in mental health outcomes since the Better Access Scheme (Jorm, 2018; Skinner et al., 2022). Some studies, rather, observed a trend of steady and significant growth in the prevalence of clinically significant psychological in recent years in Australia (Butterworth et al., 2020; Enticott et al., 2022). In specific, a substantial increase from 3.5 to 7.2% in very-high level psychological distress and from 12.4 to 18.7% in high/very-high level psychological distress was recorded respectively based on an analysis on Australia’s six consecutive NHS from 2001 to 2017-18 (Enticott et al., 2022). Nevertheless, other explanations for such phenomenon should also be considered, including 1) mental health surveys could only provide rough estimations of the prevalence of mental health disorders, and they typically do not measure incidence and may not be able to separate active mental disorders from chronic and controlled conditions; 2) mental health treatments can barely cut the lifetime prevalence of mental disorders, and limited efficacy of treatments can also contribute to the lack of decrease in mental disorders rates (Furukawa and Kessler, 2019). Moreover, wealthier individuals are more likely to access a greater number of psychological sessions and can afford to continue utilizing mental health services beyond the limits of Medicare-subsidised sessions, in contrast to their less-wealthy counterparts (Dawadi et al., 2024). Correspondingly, a study using dynamics model analysis of mental disorder incidence and treatment-related recovery in Australia from 2008 to 2019 found increase in personal-level risks of developing high and very-high psychological distress that might have close connection with the increase to the increased mental disorders rate and they discovered no evidence of decrease in treatment effectiveness (Skinner et al., 2022).

**Supplementary Material S11.** Possible responses

There is a major task to get evidence-based care to the population and empowering recovery- oriented ways in the population, as this is the evidence-based paradigm (Meadows et al., 2019). Elsewhere work has highlighted the rapid increase in poor mental health in Australian women aged 55-64 (Enticott et al., 2022) who also are at high risk of unemployment, poverty and homelessness, and as supported by the Lancet global mental health commission, addressing social determinants is also key to improving population mental health (Patel et al., 2018). It has been suggested that the nature of the challenge facing mental health globally is so extreme that the existing evidence and investigative paradigm should be exchanged radically for very different service models (van Os et al., 2019). We remain optimistic then that care along the lines of available mental health care models especially including a recovery orientation, if underpinned by evidence and widely available, could serve to appreciably influence mental health of populations, including Australia’s.

**References:**

Australian Institute of Health and Welfare (AIHW) (2020-21) *Expenditure on mental health-related services*. Available at: <https://www.aihw.gov.au/mental-health/topic-areas/expenditure> (accessed Nov 2).

Butterworth P, Watson N and Wooden M (2020) Trends in the Prevalence of Psychological Distress Over Time: Comparing Results From Longitudinal and Repeated Cross-Sectional Surveys. *Frontiers in Psychiatry* 11.

Dawadi S, Shawyer F, Callander E, et al. (2024) An equity indicator for assessing mental healthcare access: a national population case study. *Epidemiology and psychiatric sciences* 33: e70.

Department of Health and Aged Care (DoHAC) (2023) *Better Access initiative*. Available at: <https://www.health.gov.au/our-work/better-access-initiative> (accessed Nov 2).

Enticott J, Dawadi S, Shawyer F, et al. (2022) Mental Health in Australia: Psychological Distress Reported in Six Consecutive Cross-Sectional National Surveys From 2001 to 2018. *Frontiers in Psychiatry* 13.

Furukawa TA and Kessler RC (2019) Why has prevalence of mental disorders not decreased as treatment has increased? *Australian and New Zealand Journal of Psychiatry* 53(12): 1143-1144.

Jorm AF (2018) Australia’s ‘Better Access’ scheme: Has it had an impact on population mental health? *Australian and New Zealand Journal of Psychiatry* 52(11): 1057-1062.

Looi JCL, Maguire PA, Allison S, et al. (2022) Medicare-subsidised mental health services from the beginning of Better Access in 2006–2007 to 2019–2020: Descriptive analysis by state, profession and consultation profile. *Australasian Psychiatry* 30(5): 640-652.

Meadows GN, Prodan A, Patten S, et al. (2019) Resolving the paradox of increased mental health expenditure and stable prevalence. *Australian and New Zealand Journal of Psychiatry* 53(9): 844-850.

Patel V, Saxena S, Lund C, et al. (2018) The Lancet Commission on global mental health and sustainable development. *The Lancet* 392(10157): 1553-1598.

Pirkis J, Currier D, Harris M, et al. (2022) Evaluation of the Better Access initiative. Reportno. Report Number|, Date. Place Published|: Institution|.

Skinner A, Occhipinti JA, Song YJC, et al. (2022) Population mental health improves with increasing access to treatment: evidence from a dynamic modelling analysis. *BMC Psychiatry* 22(1): 692.

Sun X, Briel M, Walter SD, et al. (2010) Is a subgroup effect believable? Updating criteria to evaluate the credibility of subgroup analyses. *Bmj* 340.

van Os J, Guloksuz S, Vijn TW, et al. (2019) The evidence-based group-level symptom-reduction model as the organizing principle for mental health care: time for change? *World Psychiatry* 18(1): 88-96.

Whiteford HA, Buckingham WJ, Harris MG, et al. (2014) Estimating treatment rates for mental disorders in Australia. *Australian Health Review* 38(1): 80-85.

1. The study inspected the primary purposes, topics, placement of questions, target populations, inclusion and exclusion criteria, methodologies, and statistical techniques of the three surveys. Except the variables of health service use and long-term mental health conditions, main variables used in this study were all directly or moderately comparable across the three NHS. [↑](#footnote-ref-1)
2. Medications recorded in the “General Medication Use” variable were coded according to the World Health Organisation (WHO) Anatomical Therapeutic Chemical (ATC) classification system. [↑](#footnote-ref-2)
3. The “Long-Term Mental Health Conditions” variable is a binary variable containing two categories as “Yes” and “No”, indicating whether a person reported to have current and long-term (that had lasted, or was expected to last, for 6 months or longer) mental health conditions. The long-term mental health conditions included mood disorders, such as “feeling depressed”, “other mood (affective) disorders” and “depression”, and anxiety related problems, such as “feeling anxious, nervous or tense”, “anxiety disorders”, “panic disorders/panic attacks”, “phobic anxiety disorders”, “post-traumatic stress disorder”, and “obsessive compulsive disorder”.Conditions recorded in this variable were classified based on the 10^th^ Revision of the International Classification of Diseases (ICD10). [↑](#footnote-ref-3)
4. The “Health Service Use” variable is a binary variable containing two categories as “Yes” and “No”, indicating whether a person has consulted with a GP or psychologist for health in the last 12 months. [↑](#footnote-ref-4)
5. The “Physical Comorbidity” variable includes categories of no comorbidity, only 1 comorbidity, 2 - 4 comorbidities, and 5 or more comorbidities, which include the conditions of diabetes, kidney disease, cardiovascular disease, arthritis, other musculoskeletal conditions, asthma, and other chronic lower respiratory disease that were classified based on the ICD10. [↑](#footnote-ref-5)
6. The “Geographical Location” variable recorded remoteness area categories of participants’ households, which were categorised into major cities of Australia, inner regional Australia, and other. In the 2011-12, 2014-15, and 2017-18 NHS, the remoteness area categories employed the Australian Statistical Geography Standard (ASGS) instead of the ASGC Remoteness classification based on the Accessibility/Remoteness Index of Australia (ARIA+) as used in previous surveys. [↑](#footnote-ref-6)
